# New Amidino-Substituted Benzimidazole Derivatives as Human Dipeptidyl Peptidase III Inhibitors: Synthesis, In Vitro Evaluation, QSAR, and Molecular Docking Studies

**DOI:** 10.3390/ijms26083899

**Published:** 2025-04-20

**Authors:** Dejan Agić, Maja Karnaš Babić, Marijana Hranjec, Domagoj Šubarić, Zrinka Karačić, Marija Abramić

**Affiliations:** 1 Faculty of Agrobiotechnical Sciences Osijek, Josip Juraj Strossmayer University of Osijek, 31000 Osijek, Croatia; maja.karnas@fazos.hr (M.K.B.); domagoj.subaric@fazos.hr (D.Š.); 2 Department of Organic Chemistry, Faculty of Chemical Engineering and Technology, University of Zagreb, 10000 Zagreb, Croatia; mhranjec@fkit.unizg.hr; 3 Division of Molecular Biology, Ruđer Bošković Institute, 10000 Zagreb, Croatia; zrinka.karacic@irb.hr; 4 Division of Organic Chemistry and Biochemistry, Ruđer Bošković Institute, 10000 Zagreb, Croatia; marija.abramic@irb.hr

**Keywords:** dipeptidyl peptidase III inhibitor, metallopeptidase family M49, amidines, benzimidazoles, QSAR, molecular docking

## Abstract

Dipeptidyl peptidase III (DPP III) is a zinc-dependent enzyme that hydrolyses biologically active peptides by cleaving dipeptides from their amino terminus. While the fundamental role of this metallopeptidase remains incompletely understood, human DPP III (hDPP III) has been linked to several pathophysiological processes relevant to drug development. In this study, thirty-six amidino-substituted benzimidazole derivatives, including seven newly synthesized compounds, were examined for their activity against hDPP III by combining in vitro tests, in silico quantitative structure–activity relationship (QSAR) modelling, and molecular docking approaches. The experiments demonstrate that all compounds display inhibitory activity at a 30 µM concentration. A biochemical assay revealed that 2,2′-bithiophene, 4-trifluoromethylphenyl, 4-(*N,N*-diethylamino)phenyl, and 2,3,4-trihydroxyphenyl as substituents at position 2 of the benzimidazole core enhance inhibitor potency. Additionally, the type of substituent at positions 5(6) of the benzimidazole core influences enzyme inhibition, with effectiveness ranked as follows: 2-imidazolinyl > unsubstituted amidine > 2-tetrahydropyrimidine. A multiple linear regression QSAR model for hDPP III inhibition was developed using four Dragon descriptors (*Rww*, *Mats3e*, *BELe4*, and *nCs*), which can explain 82% of the inhibitory activity. Docking analysis of the semi-closed form of hDPP III in a complex with the most potent compounds indicates the structural features of the benzimidazole derivatives important for the binding at the hDPP III active site.

## 1. Introduction

Dipeptidyl peptidase III (DPP III; DPP3; EC 3.4.14.4) is a cytosolic, widely distributed mono-zinc peptidase that catalyses the cleavage of a dipeptide from the unsubstituted amino-end of its substrates. DPP III shows broad specificity towards peptides, optimally consisting of four to eight amino acids, but prefers the diarginyl arylamides among the synthetic substrates [1]. Biochemical studies in vitro have demonstrated a high affinity of mammalian DPP III for a number of biologically active peptides [1,2,3,4], indicating that, besides the participation in the final steps of intracellular protein catabolism, more specific biological roles could be ascribed to this protease.

**Figure 1:**
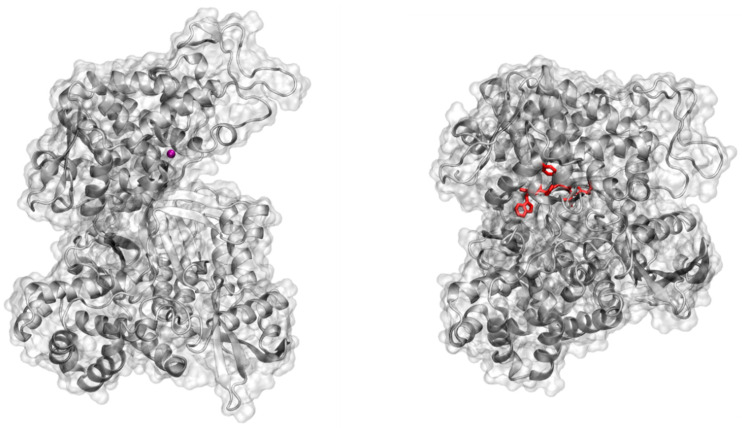
Representation of the secondary structural elements with a transparent molecular surface of ligand-free human DPP III (PDB code: 3fvy) (**left**) and its complex with pentapeptide tynorphin (PDB code: 3t6b) (**right**). The zinc ion is a purple sphere, while the pentapeptide is coloured red.

While eukaryotic DPP IIIs, mostly mammalian, were isolated and extensively experimentally characterized first [1], bacterial DPP IIIs were discovered in silico through database searches and multiple sequence alignments, thus enabling the recognition of the unique DPP III family (metallopeptidase family M49) [5,6]. The crystal structure of yeast and human ligand-free DPP III revealed an elongated protein with two lobe-like domains separated by a wide cleft [7,8]. The zinc-binding site and the catalytically important amino acid residues are located in the “upper” lobe. The 3-D structure of human DPP III with ligand bound and long molecular dynamics (MD) simulations showed a huge domain motion and the complete closure of the cleft around the bound peptide substrate (Figure 1) [8,9].

The increased research interest in mammalian DPP III in the last decade has provided evidence for several (patho)physiological roles. Among the most important are the involvement of DPP III in the degradation of the bioactive peptides in the renin–angiotensin system (RAS), which is physiologically relevant for blood pressure regulation, and its participation in the endogenous defence against oxidative stress [10,11]. DPP III binds to the Keap 1 protein, a constituent of the Keap 1-Nrf2 signalling pathway, the cell’s main defence mechanism against extrinsic and intrinsic oxidative and electrophilic stimuli. Consequently, transcription factor Nrf2 is displaced from Keap 1 and translocated to the nucleus, initiating the expression of genes that encode cytoprotective antioxidant enzymes [11].

Enhanced expression of DPP III has been reported in different malignant tissues and is correlated with poor prognosis in patients with breast cancer, colorectal and oesophageal carcinoma, lung squamous cell carcinoma, and multiple myeloma [12,13,14,15,16]. A correlation between DPP III activity and aggressiveness was found previously in ovarian carcinomas [17]. Recent studies revealed that the pathophysiological role of DPP III in malignant growth could be connected to its interaction with the Keap 1-Nrf2 pathway [12] and cyclin-dependent kinase 1 (CDK1) [13].

As a cytosolic enzyme, DPP III is released into the circulation upon cell death. Most recent clinical investigations have shown that circulating DPP III plasma concentration (cDPP3) increases under conditions of cardiogenic shock and sepsis [18,19]. Furthermore, circulating DPP III has been reported as an early biomarker for the severity of cardiogenic shock, correlated with high mortality risk [20]. Similarly, the highest levels of cDPP3 were found in non-surviving septic shock patients [19]. Altogether, these findings on the pathological significance identified human DPP III as a potential drug target that could improve outcomes of disease through its selective inhibition [21].

To date, known inhibitors of DPP III belong to various classes of mostly small molecular weight compounds of natural or synthetic origin [22]. A small number of cyclobutane derivatives containing amidino-substituted benzimidazole moieties have been examined earlier as potential inhibitors of human DPP III hydrolytic activity. It was shown that the amidino group placed at the termini of the molecules enhanced inhibitory potential towards this enzyme [23].

Taking into consideration the great biological importance and versatile pharmacological features of natural, semisynthetic, or synthetic benzimidazole derivatives, this nitrogen subunit has become an unavoidable structural motif in the rational design of novel drugs [24,25]. Benzimidazole derivatives display a broad spectrum of biological activities, including anticancer, antiviral, antioxidant, antibacterial, antifungal, antihistaminic, and anti-inflammatory effects [26,27,28]. Furthermore, their biological significance is recognized due to the similarity of the benzimidazole nucleus with naturally occurring purines, which allows the interaction of benzimidazole derivatives with polynucleotides such as DNA and/or RNA [29,30]. On the other hand, amidines, important nitrogen analogues of carboxylic acids, are well-known pharmacophores that occur as structural parts of numerous biologically active molecules, improving their biological features [31]. Compounds substituted with an amidine moiety possess an extensive range of versatile biological activities [32]. Amidine groups contribute significantly to the formation of complexes between the active molecule and possible biological target, mostly through H-bonding and electrostatic interactions [33]. Since the amidine group is usually placed at the end of the molecule in the cationic form, it allows the orientation of the molecule toward the binding to an electronegatively charged molecule [34].

As a continuation of our interest in inhibitors of human DPP III, we present here the investigation of 36 benzimidazole derivatives, including seven newly synthesized compounds bearing three types of amidine substituents: unsubstituted amidine, 2-imidazolinyl, or 2-tetrahydropyrimidinyl. The relationship between the inhibitory profile of the benzimidazole derivatives towards hDPP III was investigated through an in vitro assay alongside Quantitative Structure–Activity Relationship (QSAR) modelling. Moreover, we provide a detailed analysis of the non-covalent interactions between the semi-closed form of hDPP III and the most potent inhibitors utilizing a molecular docking study.

## 2. Results and Discussion

### 2.1. Chemistry

**Scheme 1:**
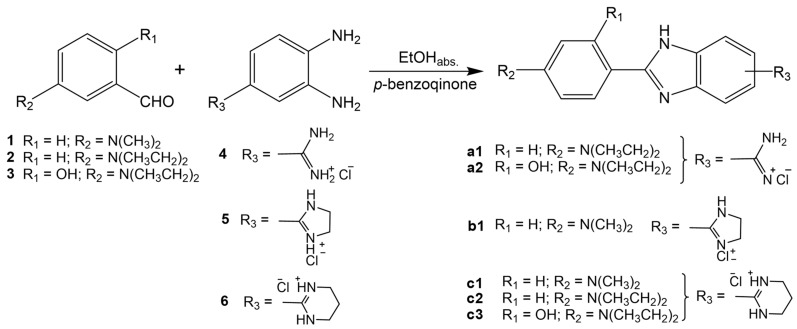
Preparation of amidino substituted benzimidazoles **a1**, **a2**, **b1**, **c1**, **c2,** and **c3**.

**Scheme 2:**
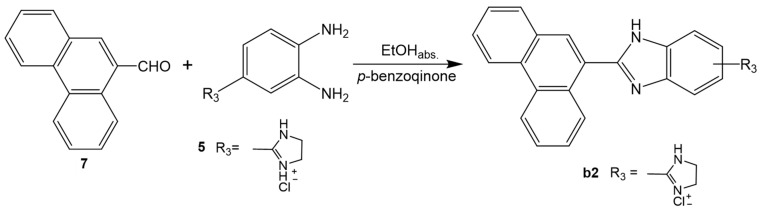
Preparation of amidino-substituted benzimidazole **b2**.

Seven new amidino-substituted benzimidazole derivatives—**a1**, **a2**, **b1**, **b2**, **c1**, **c2**, and **c3**—were synthesized according to the experimental procedures outlined in Scheme 1 and Scheme 2. The preparation and characterization of the other benzimidazole derivatives used in this study (**a3**–**a12**, **b3**–**b13**, and **c4**–**c11**) have been published previously [35,36,37,38,39]. Cyclocondenastion, which leads to the fused benzimidazole derivatives, was performed using conventional and previously described synthetic methods, starting from the corresponding benzaldehydes **1–3** and phenanthrene-9-carboxaldehyde **7** [35]. Within the reaction of cyclocondensation, the desired amidino-substituted benzimidazoles were obtained in moderate reaction yields from the aforementioned aryl aldehydes **1–3** and previously prepared amidino-substituted 1,2-phenylenediamines **4–6**, using p-benzoquinone as an oxidant. Amidino-substituted intermediates **4–6** were obtained in the acidic Pinner reaction from the corresponding cyano-substituted precursors, according to previously published and optimized reaction procedures [36]. To enhance their water solubility, all amidino-substituted derivatives were prepared as hydrochloride salts.

The structures of newly synthesized amidino-substituted benzimidazoles were confirmed using ^1^H and ^13^C NMR spectroscopy. NMR analysis, based on the values of chemical shifts and H-H coupling constants in the ^1^H spectra, confirmed the structures of the compounds. The ^13^C NMR chemical shifts were consistent with the suggested structures. In addition, IR spectroscopy was used to monitor the Pinner reaction due to the synthesis of main precursors **4–6**. The observation of the signal related to the NH group of benzimidazole nuclei directly confirmed successful cyclocondensation. Amidino group signals appear around 10 ppm, while protons from cyclic amidino substituents are seen in the aliphatic region of the 1H NMR spectrum for unsubstituted compounds and in both ^1^H and ^13^C NMR for substituted ones (Supplementary Figures S1–S14).

### 2.2. Human DPP III Inhibitory Activity

**Figure 2:**
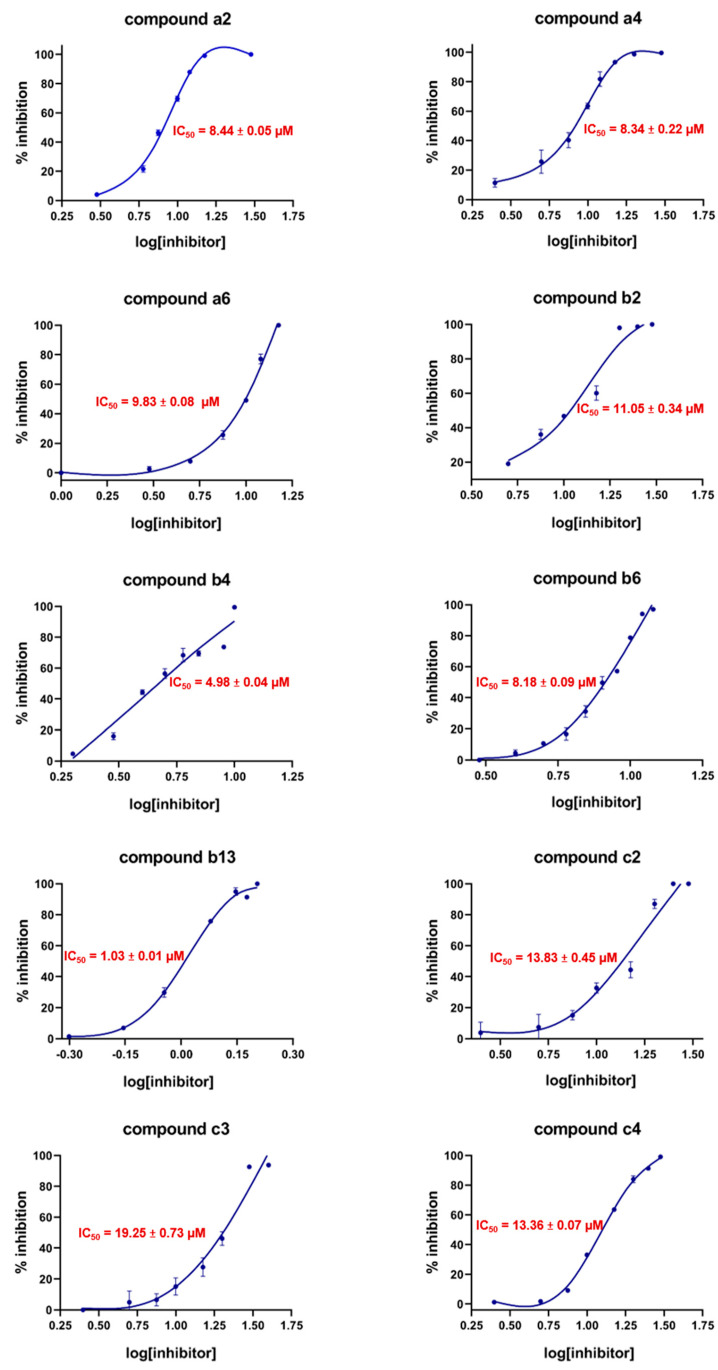
IC_50_ curves of compounds **a2**, **a4**, **a6**, **b2**, **b4**, **b6**, **b13**, **c4**, and **c3** against human DPP III. Dose–response data points represent the mean value ± SD of three replicates.

This study investigated 36 amidino-substituted benzimidazole derivatives for their in vitro inhibitory potential against human dipeptidyl peptidase III (hDPP III). Each compound exhibited inhibitory activity at a concentration of 30 µM; however, potency variations were observed among the derivatives, as detailed in Table 1. To provide a better overview and more efficient discovery of structure–activity relationship trends, the compounds were categorized into three groups based on the substituent at position 5(6) of the benzimidazole core: unsubstituted amidine derivatives (labelled as **a**), 2-imidazolinyl derivatives (labelled as **b**), and tetrahydropyrimidine derivatives (labelled as **c**). The strongest inhibition (% inh. ≥ 90) was observed with compounds **a2**, **a4**, **a6**, **b2**, **b4**, **b6**, **b13**, **c2**, **c3**, and **c4**, all of which demonstrated IC_50_ values below 20 μM (Figure 2). A slightly less effective inhibition (% inh. ≥ 80 to < 90) was obtained for compound **a9**, while compounds **a1**, **a3**, **a5**, **a12**, **b1**, **b3**, **b7**, **b9**, and **c8** showed moderate inhibition (% inh. ≥ 50 to <80). Compounds **a7**, **a8**, **a10**, **b5**, **b8**, **b10**, **b11**, **b12**, **c7**, and **c11** showed mild inhibition (% inh. ≥ 20 to <50), whereas compounds **a11**, **c1**, **c5**, **c6**, **c9**, and **c10** showed weak inhibitory effects (% inh. < 20).

When comparing the relationship between the structures and inhibitory activity of the tested compounds, it is evident that 2,2′-bithiophene-derived benzimidazole derivatives, **b4**, **a4**, and **c4**, demonstrated a high percentage of enzyme inhibition, with IC_50_ values of 4.98 ± 0.04 μM, 8.34 ± 0.22 μM, and 13.36 ± 0.07 μM, respectively (Figure 2). Furthermore, IC_50_ values indicate that the 2-imidazolinyl group at position 5(6) of the benzimidazole core enhances the inhibitory effect more effectively than their amidine and tetrahydropyrimidine analogues. The type of substituent at position 5(6) similarly affected the antiproliferative activity of **b4**, **a4**, and **c4** against human cancer cell lines, including HCT116 (colon carcinoma), MCF-7 (breast carcinoma), H460 (lung carcinoma), and PC3 (prostate carcinoma) [36].

The benzo[*b*] thiophene-derived benzimidazole derivatives **a3**, **b3**, and **c11** exhibit moderate to mild inhibitory activity. In the first two compounds, despite the presence of different groups (**a** and **b**) at position 5(6) of the benzimidazole core, their influence on enzyme inhibition was minimal, with inhibition levels recorded at 60.6% and 59.4%, respectively. When the tetrahydropyrimidine analogue (**c11**) occupied the same position, there was a decrease in inhibition, dropping to 28.9%.

Two of three 4-trifluoromethylphenyl-derived benzimidazole derivatives exhibited nearly complete inhibition of enzyme activity at 30 µM concentration. Compound **b6** demonstrated an IC_50_ value of 8.18 ± 0.09 μM. In comparison, compound **a6** also exhibited strong activity but with a slightly lower inhibition potential, showing an IC_50_ of 9.83 ± 0.08 μM. Both derivatives displayed antiproliferative activity against human cancer cell lines HCT116, MCF-7, and H460. It was noted that the imidazolinyl group enhances this activity compared to the amidino group [39]. The presence of tetrahydropyrimidine at position 5(6) of compound **c6** resulted in a reduction of inhibitory activity, which was 15.5% at a concentration of 30 µM (Table 1).

The impact of various substituents at position 5(6) on the inhibitory activity is prominently demonstrated in three 4-cyanophenyl-derived benzimidazole derivatives. Compound **a5** achieved a moderate inhibition of 67.8%, while **b5** and **c5** showed an inhibition of 34.4% and 0.1%, respectively. Similar to compound **c5**, its phenyl-derived benzimidazole analogue **c9** showed a weak inhibitory potential of 2.1%.

Among the three 3*H*-benzimidazolyl-derived benzimidazole derivatives, **b7** demonstrated the highest activity with a 51.5% inhibition rate. In contrast, **c7** and **a7** exhibited mild inhibitory activities of 29.8% and 21.6%, respectively, when tested at 30 µM (Table 1).

The presence of the 4-(*N*,*N*-diethylamino) phenyl group at position 2 of the benzimidazole core in the newly synthesized compounds **c2**, **a1**, **a2**, and **c3** contributed largely to their higher inhibitory activity. The substituents at position 6 in **c2** and **a1** reveal interesting differences in their effects on enzyme inhibition. In **c2**, the presence of the tetrahydropyrimidine group completely inhibited the enzyme at a concentration of 30 µM, with an IC_50_ value of 13.83 ± 0.45 μM. In contrast, the amidino group at position 5(6) in compound **a1** showed moderate inhibition of 62.9%. The hydroxylation of the 4-(*N*,*N*-diethylamino) phenyl group at position 2 in **a1** and **c2** resulted in the complete inhibition of the enzyme by analogue **a2** (IC_50_ of 8.44 ± 0.05 μM) and relatively weaker inhibitory capacity by analogue **c3** (IC_50_ of 19.25 ± 0.73 μM) (Figure 2). The substitution of the 4-(*N*,*N*-diethylamino)phenyl group at position 2 of the benzimidazole core with a 4-(*N*,*N*-dimethylamino)phenyl group led to a decrease in the inhibitory activity of compound **c1**. Specifically, the activity was reduced by approximately 90% compared to its analogue, compound **c2**. In contrast, 4-(*N*,*N*-dimethylamino) phenyl-derived benzimidazole **b1** exhibited moderate inhibition of 52.9%.

Both naphthalene-1-yl-derived benzimidazole derivatives, **a8** and **b8**, exhibited mild inhibition of 38.7% and 29.5%, respectively. In comparison, the enzyme inhibition by the naphthalene-2-yl-derived benzimidazole derivative **c10** was only 8.0%. The newly synthesized phenanthrene-9-yl-derived benzimidazole derivative **b2** demonstrated complete inhibition of the enzyme, with an IC_50_ value of 11.05 ± 0.34 μM, while its analogue **c8** exhibited almost half the inhibitory effect on hDPP III (51.7%).

Quinolin-2-yl-derived benzimidazole derivative **a9** exhibited a slightly stronger inhibitory effect compared to its analogue **b9**, which has a 2-imidazolinyl group at position 5(6) of the benzimidazole core (82.9% versus 73.4% inhibition).

The presence of the 2-hydroxy-4-methoxyphenyl group at position 2 of the benzimidazole core in **a12** and **b12** resulted in approximately 50% inhibition of the enzyme. The amidino group at position 6 in compound **a12** had a minimal effect on enhancing the inhibitory activity compared to the 2-imidazolinyl group at the same position in compound **b12** (Table 1).

The influence of different groups at position 5(6) on inhibition was also demonstrated in benzimidazole derivatives coupled to 2-hydroxyphenyl and 2,4-dihydroxyphenyl. The 2-imidazolinyl group in **b10** and **b11** exhibited a stronger inhibitory effect (42.8% and 41.1% of inhibition, respectively) compared to their amidino analogues **a10** and **a11**, which showed inhibition of 24.3% and 14.6%, respectively. The hydroxylation at position 3 of the phenolic group is essential for effective hDPP III inhibition. This was demonstrated by 2,3,4-trihydroxyphenyl benzimidazole in **b13**, which exhibited the strongest inhibitory effect among all the tested compounds, with an IC_50_ value of 1.03 ± 0.01 µM (Figure 2).

The analysis of the relative occupancy of enzyme inhibition in compounds with the same substituent at position 5(6) of the benzimidazole core also helped to obtain insights into the structure–activity relationships. The results of this analysis indicate that compounds with the 2-imidazolinyl group exhibit the highest occupancy (0.59), followed by those containing an amidino group (0.56), while compounds with a tetrahydropyrimidine substituent show the lowest relative occupancy (0.36). In addition, more than half of the compounds in group **b** (8 out of 13) or group **a** (8 out of 12) exhibited strong to moderate inhibition. However, only four out of 11 compounds in group **c** showed similar inhibitory potency.

**Figure 3:**
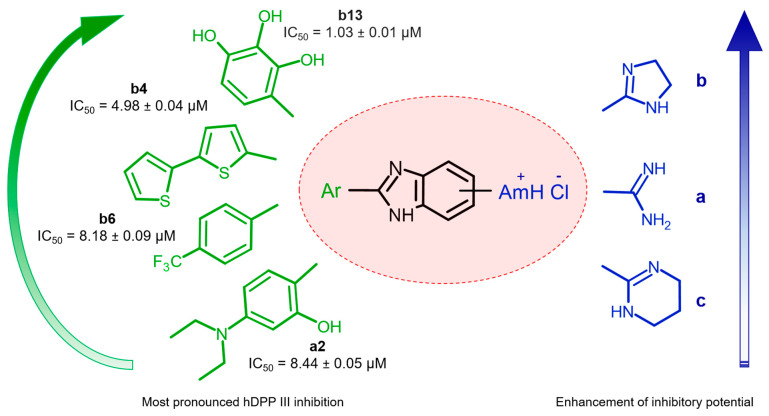
Structure–activity relationships of amidino-substituted benzimidazole derivatives toward human DPP III.

The data presented above reveal that certain substituents play a critical role in enhancing the inhibitory effectiveness of tested benzimidazole derivatives. In particular, substituents such as 2,2′-bithiophene, 4-trifluoromethylphenyl, 4-(*N*,*N*-diethylamino)phenyl, and 2,3,4-trihydroxyphenyl at position 2 of the benzimidazole core mostly improve inhibitor potency, with an IC_50_ value below 10 µM. Furthermore, the type of substituent at position 5(6) mainly affected enzyme inhibition, ranking in effectiveness as follows: 2-imidazolinyl (group **b**) > amidine (group **a**) > 2-tetrahydropyrimidine (group **c**) (Figure 3). The results of this in vitro study are in line with our earlier experiments involving a small set of four benzimidazole derivatives, where we reported that the cyclization of the amidino group into the imidazolinyl group enhanced the inhibitory potential against hDPP III [23].

### 2.3. QSAR Model Analysis

The best multiple linear regression QSAR model obtained for hDPP III inhibition by benzimidazoles is:

(1) log⁡% hDPP III inh.=−3.90+0.06Rww+1.25Mats3e+3.81BELe4−0.94nCs 

where *Rww* is the reciprocal hyper-detour index, *Mats3e* is a Moran autocorrelation of lag 3 weighted by atomic Sanderson electronegativities, *BELe4* lowest eigenvalue number 4 of the Burden matrix weighted by atomic Sanderson electronegativities, and *nCs* is the number of total secondary sp^3^ carbon atoms. The variables in the model equation are listed in order of their relative importance by the standardized regression coefficient. The logarithmic values of hDPP III inhibition percentage, experimentally obtained and calculated by the model equation, are given in Table 1. The values of the descriptors included in the model for all compounds are given in Supplementary Table S1. Logarithmic values calculated by the model equation that slightly exceed 2.00 indicate that these compounds could be potent inhibitors even at lower concentrations.

The statistical parameters for this model are presented in Table 2. The collinearity of the descriptors in the model was assessed using the correlation matrix (Supplementary Table S2), which indicated a minimal risk of overfitting, as all correlation coefficients were below 0.70. Furthermore, the values of the multivariate correlation index (*Kxx*) and the global correlation among descriptors (*ΔK*) provide additional evidence that there is no linear relationship among the descriptors [40].

The model satisfied the threshold values for fitting and internal validation criteria [41]. Based on the coefficient of determination (*R*^2^), this model can explain 82% of the inhibitory effect of benzimidazoles on hDPP III. The model’s stability and robustness were confirmed through cross-validation using both the leave-one-out (LOO) and leave-many-out (LMO) procedures. The cross-validated explained variances (*Q*^2^*_LOO_* and *Q*^2^*_LMO_*) are both higher than 0.6. Furthermore, the coefficients obtained from the Y-scramble method, the correlation coefficient (*R*^2^*_Yscr_*), and the cross-validation coefficient (*Q*^2^*_Yscr_*) are both less than 0.02, indicating that the model was not developed by chance [42,43]. External validation confirmed the model’s predictive ability: *R*^2^ for the external set is greater than 0.60; the concordance correlation coefficient (*CCC_ext_*) for the test set is above 0.80; and both the root-mean-square error (*RMSE_ext_*) and mean absolute error (*MAE_ext_*) are low. Finally, all predictive squared correlation coefficients (*Q*^2^*_Fn_*) are higher than 0.60 [41].

**Figure 4:**
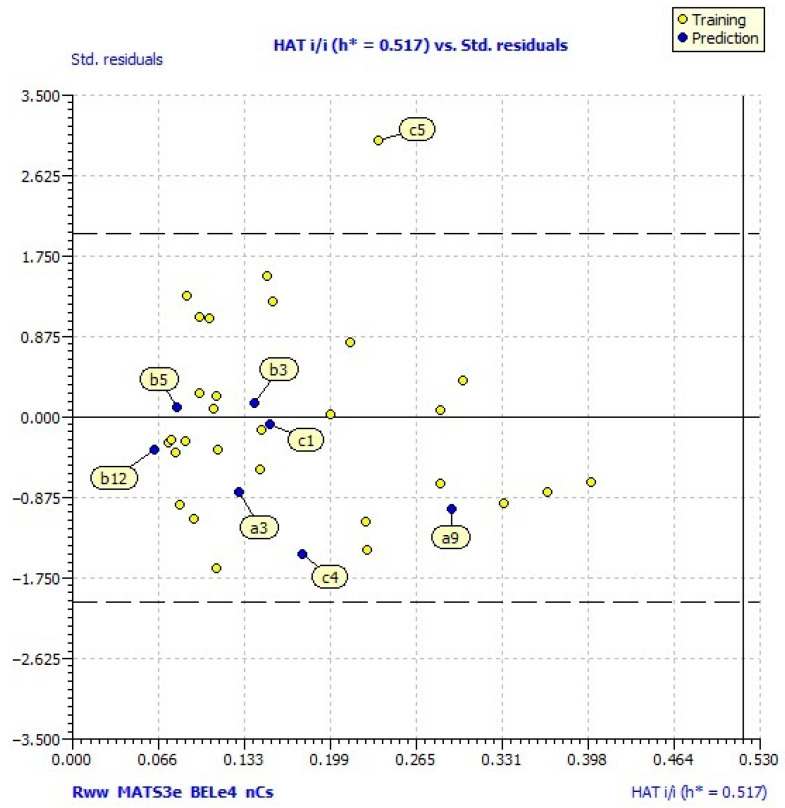
Williams plot of the applicability domain of the QSAR model (Equation (1)).

The applicability domain analysed using the Williams plot (Figure 4) indicated that all compounds fall within the warning leverage threshold (h* = 0.517). Compound **c5** was identified as an outlier because its cross-validated standardized residual exceeds two standard deviation units. However, excluding this compound did not enhance the quality of the model. The variables from the best model were more closely observed to reveal the factors contributing to the inhibitory activity of tested benzimidazoles. The signs of the regression coefficients in the model equation indicate how individual descriptors influence the model. A positive regression coefficient linked to a descriptor will enhance the activity profile of a compound, while a negative coefficient will have a detrimental effect. The first variable in the model equation, descriptor *Rww*, represents the reciprocal hyper-detour index, which is a topological descriptor. Topological descriptors are derived from a graph representation of the molecule and serve as numerical quantifiers of molecular topology [44]. They are obtained by applying algebraic operators to matrices that represent molecular graphs, and their values remain consistent regardless of vertex numbering or labelling [45].

They are obtained by applying algebraic operators to matrices that represent molecular graphs, and their values remain consistent regardless of vertex numbering or labelling [45]. These descriptors can be sensitive to various structural features of a molecule, such as size, shape, symmetry, branching, and cyclicity. They can also encode chemical information related to atom types and bond multiplicity [46]. The positive regression coefficient of the *Rww* descriptor suggests that a higher value of this descriptor would be advantageous for the compound’s inhibitory activity.

*Mats3e* belongs to the 2D-autocorrelation descriptors, calculated from the molecular graph by summing the products of atom weights of the terminal atoms of all the paths of the considered path length (the lag). For spatial autocorrelation molecular descriptors calculated on a molecular graph, the lag coincides with the topological distance between any pair of vertices (atoms). *Mats3e* is weighted by Sanderson electronegativity, positive autocorrelation corresponds to positive values of this coefficient, whereas negative autocorrelation gives negative values [44]. All compounds except **b13** show negative values of *Mats3e*; however, the most active compounds tend to have fewer negative values of this descriptor. Therefore, the distribution of electronegative atoms at the topological distance of 3 could be beneficial for the enhanced inhibitory activity against hDPP III. On the other hand, descriptor *BELe4*, which is also weighted by electronegativity, is part of the BCUT (Burden—CAS—University of Texas eigenvalues) group of molecular descriptors derived from the positive and negative eigenvalues of the adjacency matrix. These descriptors represent an extension of the Burden approach. The Burden parameters combine the atomic number for each atom with a description of the nominal bond types for both adjacent and non-adjacent atoms. The BCUT metrics broaden the range and types of atomic features by considering three classes of matrices, where the diagonal elements correspond to atomic charge-related values, atomic polarizability-related values, and atomic hydrogen bond abilities [44,47].

*BELe4* in the model equation bares a positive coefficient which, together with the positive values of this descriptor for all compounds, means its enhanced values contribute to the inhibition of hDPP III. Furthermore, both *Mats3e* and *BELe4* descriptors might be connected to water solubility, since it has been observed that as Sanderson electronegativity increases, the compound aqueous solubility also increases [48]. Three polar hydroxyl groups could therefore be the main reason for the good inhibitory activity of compound **b13**, as they contribute to the overall solubility of the compound and the possibility of hydrogen bond interactions. The presence of adequately spaced hydroxyl substituents has already been found to benefit hDPP III inhibition [49].

The last variable in the model equation, descriptor *nCs*, belongs to the functional group counts. These are simple molecular descriptors based on the quantification of specific elements within a molecule [44]. *nCs* represents the number of total sp^3^ hybridized carbon atoms (carbon atom that has sp^3^ hybridization and is bound to two other carbon atoms). This type of atom is present only in the tetrahydropyrimidine derivatives. Notably, only this descriptor has a negative correlation coefficient in the model equation, indicating that the presence of this type of carbon atom negatively impacts the inhibitory activity of benzimidazole derivatives. A similar conclusion was reached in a previous QSAR study of benzimidazole inhibitors of hDPP III. An MLR model from the study included a Kier alpha-modified shape descriptor (*S3K*) that had a negative correlation coefficient, which led to the conclusion that multiple sp^3^ hybridized atoms in benzimidazole derivatives reduce hDPP III inhibition [50].

According to the applicability domain of this model, compound **c5** is an outlier because its predicted inhibition is much higher than the observed one (Table 1). This discrepancy is mostly due to the values of the *Rww* and *BELe4* descriptors, probably because of the tetrahydropyrimidine moiety, which is larger and contains two nitrogen atoms at a topological distance of 4. The small, polar cyano substituent is considered a strong hydrogen bond acceptor and usually improves the properties of compounds it is attached to [51]. However, in the case of hDPP III inhibition, cyano-substituted derivatives do not exhibit good results [52].

**Scheme 3:**
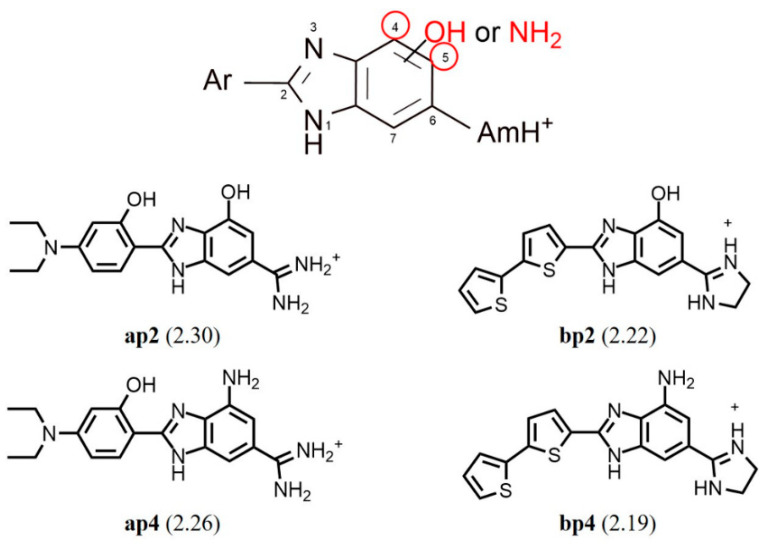
General structure of proposed benzimidazole amidines and structures of four best compounds, according to the log % inh. human DPP III, as calculated by Equation (1) (in brackets). Red circles depict C atoms on benzimidazole core suitable for substituent insertion.

Based on the information conveyed by molecular descriptors, benzimidazole amidines should be as diverse in structure as possible but avoid sp^3^ hybridized carbon atoms. Moreover, the atoms of higher electronegativity should be spaced at topological distances 3 and 4. The benzimidazole ring is thus identified as the most suitable site for the insertion of new substituents. Consequently, new structures were proposed based on the most potent compounds, determined by their IC_50_ calculations (**a2**, **a4**, **a6**, **b4**, **b6**, and **b13**). Hydroxyl and amino groups were added suitably to the benzimidazole core as depicted in Scheme 3, due to their polar nature and to maintain the desired topological distances. This modification resulted in higher values for the three most relevant descriptors, except for compound **b13**, whose analogues exhibited lower values of the *Mats3e* descriptor. Nevertheless, all the proposed compounds demonstrated high predicted inhibition of hDPP III, with the preferred substitution occurring at position 4 of the benzimidazole moiety (Supplementary Table S3). The benzimidazole analogues with the highest predicted hDPP III inhibition are the analogues of compounds **a2** and **b4**, as shown on QSAR Scheme 3.

### 2.4. Molecular Docking Study

**Figure 5:**
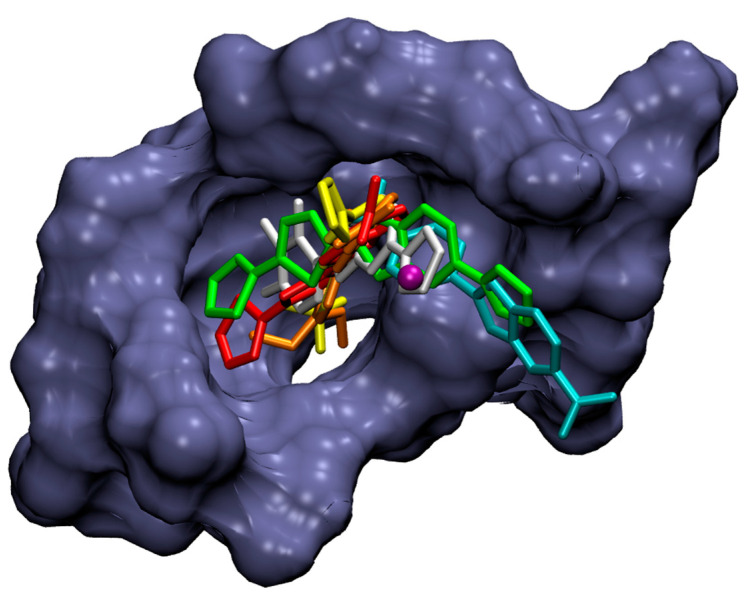
Surface representation of the overlay of the six most potent benzimidazole derivatives in the part of the central enzyme cleft of human DPP III. Compounds **a2** (orange), **a4** (red), **a6** (cyan), **b4** (green), **b6** (yellow), and **b13** (white) are shown in stick representation, and the zinc ion is presented as a purple sphere.

**Figure 6:**
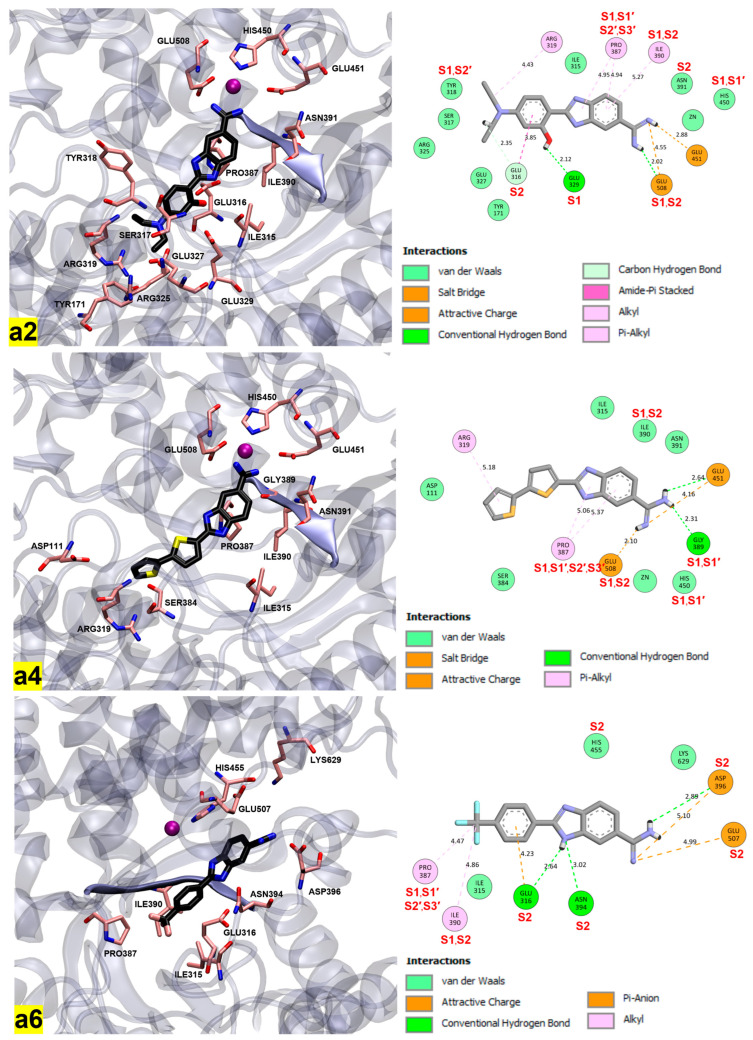

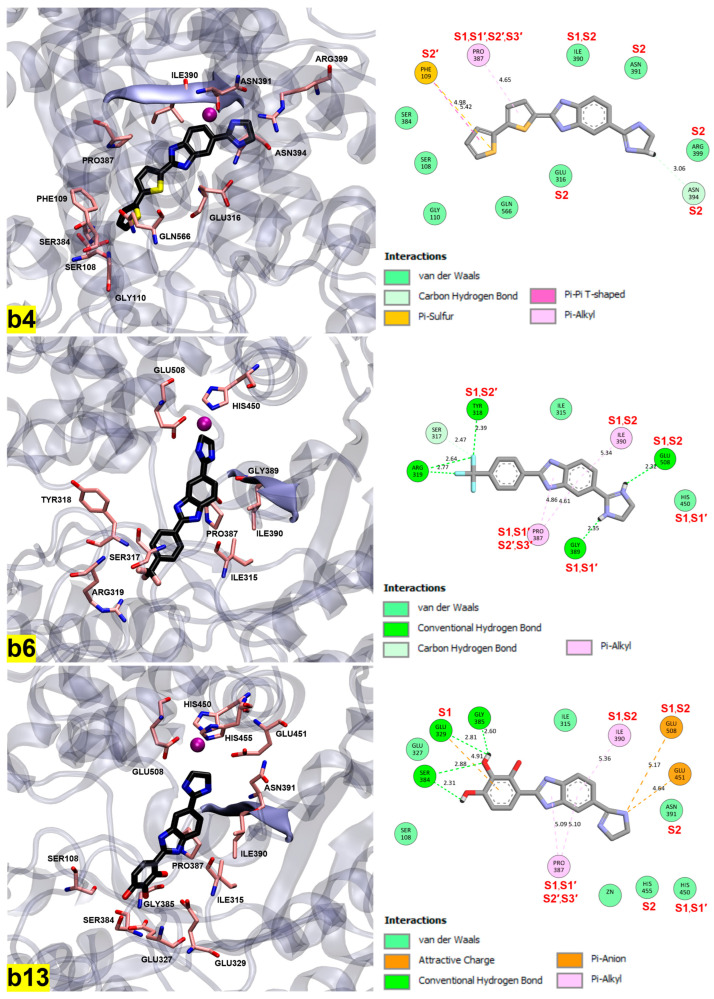
Binding of the six most potent benzimidazole derivatives at the human DPP III active site. 3D (**left**) and 2D (**right**) represent the non-covalent interactions predicted by Discovery Studio Visualizer, version 20.1.0.19295. Benzimidazole derivatives **a2**, **a4**, **a6**, **b4**, **b6**, and **b13** are shown in stick, with carbon atoms coloured black (**left**) and gray (**right**), respectively. The lower β sheet of hDPP III (residues 389–393) is coloured in ice blue, amino acid residues in pink, and the zinc ion is represented as a purple sphere. Peptide binding subsites are highlighted in red.

The study continued with molecular docking to gain insights into the potential mechanisms of inhibitory action of the six most potent benzimidazole derivatives tested in vitro, focusing on their binding affinities and the non-covalent interactions that contribute to the observed hDPP III inhibition. AutoDock Vina was employed to identify the optimal orientations of these compounds in the active site of the semi-closed form of hDPP III. The most favourable binding poses for **a2**, **a4**, **a6**, **b4**, **b6**, and **b13** were found in the area of the central enzyme cleft, near the catalytic zinc ion and the lower β-sheet (residues 389–393), with binding affinities of −7.8, −7.5, −7.7, −8.0, −8.2, and −9.0 kcal mol^−1^, respectively (Figure 5 and Figure 6). All other benzimidazole derivatives showed a similar binding mode in the active site of the semi-closed form of hDPP III, with their binding affinities listed in Supplementary Table S4.

A detailed analysis of intermolecular bond formation for the tested complexes was obtained using Discovery Studio Visualizer software, version 20.1.0.19295. Comparing the total number of interactions of the in vitro tested compounds, it is evident that the largest number of intermolecular interactions was achieved by compounds **a2** and **b13**, while others had an almost equally smaller number of interactions (Figure 6 and Supplementary Table S5). Based on the intermolecular interactions formed by various structural components of benzimidazole derivatives, the highest number of interactions occurred through their aryl and amidine groups, while the lowest number occurred through the benzimidazole core.

All six most potent compounds interacted with the enzyme binding site mostly through van der Waals forces, hydrophobic interactions, and hydrogen bonds, while attractive charge or salt bridge interactions were the least prevalent. (Figure 6 and Supplementary Table S5). Furthermore, all compounds interact with amino acid residues that are part of the peptide binding subsite [8]. Notably, they tend to interact more frequently with the S2 and S1 subsites, while interactions with the S1′, S2′, and S3′ subsites are less common (Supplementary Table S6). The analysis of interactions between various structural components of the tested benzimidazole derivatives and the peptide binding subsites reveals that the highest number of interactions occurs through the aryl and amidine groups in compounds **a2**, **a6**, **b4**, **b6**, and **b13**, relative to the benzimidazole core. In contrast, compound **a4** does not exhibit any interactions from the aryl group; however, the number of interactions from the amidine group is equal to those involving the benzimidazole core (Supplementary Table S6).

The highest binding affinity of compound **b13** is likely due to its interactions with the specific residue from the enzyme active site: the hydroxyl (OH) groups at positions 3 and 4 of the phenyl ring create four hydrogen bonds with GLU329, SER384, and GLY385; the benzimidazole core interacts with PRO387 and ILE390 through π-alkyl interactions; and the 2-imidazolinyl group forms π-anion interactions with GLU451 and GLU508, and van der Waals interaction with the catalytic zinc ion (Figure 6). Extended molecular dynamics simulations of hDPP III in complex with a coumarin derivative **12** and a quinazolinone-Schiff base **23** revealed that most of the listed amino acids contribute to their binding and stabilization in the enzyme active site [49,52]. It is important to note that GLU451 and GLU508 are part of the conserved sequence motifs ^450^H**E**LLGH^455^ and ^507^E**E**CRAE^512^, which are characteristic of members of the metallopeptidase family M49 [53]. Specifically, GLU508 coordinates the catalytic zinc atom while GLU451 serves as a general base, activating a water molecule that enables hydrolysis of the peptide bond [54]. One of the well-known potent inhibitors of hDPP III is a dipeptidyl hydroxamic acid, specifically Tyr-Phe-NHOH, which has an inhibition constant (Ki) of 0.15 ± 0.04 µM [55]. Short-term molecular dynamics simulations of the wild-type of hDPP III in complex with Tyr-Phe-NHOH demonstrated its monodentate binding through the carbonyl oxygen to the catalytic zinc ion. Additionally, the enzyme–inhibitor complex is stabilized through multiple interactions with amino acid residues that constitute the enzyme subsites S2, S1, and S1′ [56]. Among these residues are GLU451, HIS455, GLU507, and GLU508. In this study, we demonstrated that these amino acid residues contribute to the binding of compounds **a2**, **a4**, **a6**, **b6**, and **b13** to the enzyme active site.

Overall, the results of the docking study indicate that the potential mechanism of the inhibitory activity of the six most potent compounds against hDPP III could be due to multiple interactions between the aryl and amidine groups and amino acid residues that are part of the peptide binding subsite or essential for the catalytic activity of the enzyme. In contrast, the benzimidazole core appears to be less relevant in this context. This is in agreement with previous 40.8 ns long molecular dynamics simulations of hDPP III complexed with cyclobutane derivative **1**, which has two identical amidino-substituted benzimidazole moieties [50]. The stabilization of compound **1** involves the interaction between the imidazolinyl group of this compound and TYR318 and ALA416, which are part of the hDPP III peptide binding subsites S1 and S3′, respectively.

**Figure 7:**
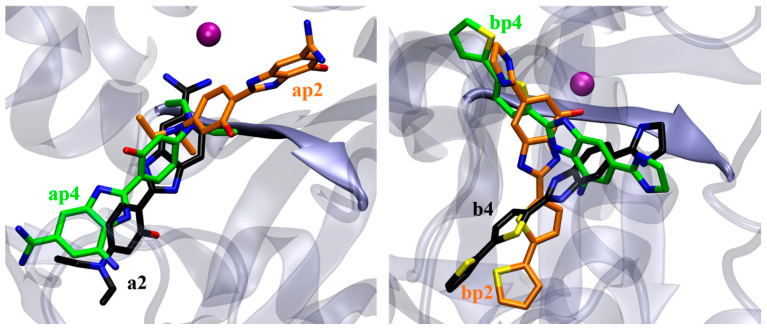
Overlay of the binding pose of **ap2** and **ap4** with their parent compound **a2** (**left**), and **bp2** and **bp4** with their parent compound **b4** (**right**) at the human DPP III active site. The zinc ion is depicted as a purple sphere, while the lower β sheet of hDPP III (residues 389–393) is coloured ice blue.

Furthermore, we conducted a molecular docking study to determine whether the QSAR-proposed compounds (Scheme 3) have affinities to form complexes with the active site of human DPP III. The most favourable binding poses for **ap2**, **ap4**, **bp2**, and **bp4** were located in the enzyme active site, with binding affinities of −8.0, −7.8, −8.4, and −8.6 kcal mol^−1^, respectively (Figure 7). The obtained binding affinity values for the proposed compounds were generally slightly higher than their parent compounds **a4** and **b4**. When comparing the positioning of **ap2** and its parent compound **a2** in the enzyme binding site, it is clear that **ap2** is placed more parallel to the β-sheet than **a2**, which is oriented antiparallel. In contrast, **ap4** occupied a similar position to **a2** but had the opposite orientation of the amidino group (Figure 7 left). In the case of **bp2**, the aryl group is positioned similarly to that of the **b4**. However, the imidazolinyl group in **bp2** is directed away from the β-sheet, while the corresponding group of **b4** points toward it (Figure 7 right).

The proposed compounds interacted with the enzyme binding site through various types of interactions, showing similar patterns to their parent compounds. However, benzimidazole cores in the proposed compounds show more interactions with hDPP III (Supplementary Table S7 and Figure S15). This is particularly evident when comparing **ap2** and **a2**, where the number of interactions was 10 and 4, respectively (Supplementary Tables S5 and S7). The substitution of a hydroxyl or amidino group at position 4 of the benzimidazole core affects their interaction (most often by hydrogen bond) with amino acids in the enzyme active site. All proposed compounds generally interact more frequently with the S2, S1, and S1′ binding subsites relative to the S2′ and S3′ subsites (Table S8 and Figure S15). Nevertheless, **bp2** and **bp4** had more interactions in the S1′ subsite than their parent compound **b4**.

## 3. Materials and Methods

### 3.1. Chemistry

#### 3.1.1. General Methods

All chemicals and solvents were purchased from commercial suppliers, including Sigma-Aldrich ( St. Louis, MO, USA) and Acros Organics (Geel, Belgium). Melting points were recorded on the SMP11 Bibby and Büchi 535 apparatus (New Castle, Delaware, USA). ^1^H and ^13^C NMR spectra were recorded on Varian Gemini 300 or Varian Gemini 600 spectrophotometers (Apeldoorn,Netherlands) at 300, 600, 75, and 150 MHz, respectively. All NMR spectra were measured in DMSO-*d_6_* solutions using TMS as an internal standard. Mass spectra were recorded with an Agilent 1100 Series LC/MSD Trap SL spectrometer (Santa Clara, CA, USA) using electrospray ionization (ESI). Chemical shifts are reported in ppm (δ) relative to TMS. All newly synthesized compounds (**a1**, **a2**, **b1**, **b2**, **c1**, **c2**, and **c3**) were routinely checked by thin layer chromatography (TLC) using precoated Merck silica gel 60F-254 plates (Rahway, NJ, USA).

#### 3.1.2. General Method for Preparation of Compounds **a1**, **a2**, **b1**, **b2**, **c1**, **c2** and **c3**

Solution of equimolar amounts of aldehydes **1**–**3** and **7**, corresponding amidines **4**–**6,** and *p*-benzoquinone in absolute ethanol were refluxed for 4 h. After the reaction mixture was cooled to room temperature, the crude product was filtered off and washed with diethyl ether. The crude product was suspended in a mixture of ethanol/diethyl ether several times until the powder was analytically pure.

**5(6)-amidino-2-4-(*N*,*N*-diethylamino)benzimidazole hydrochloride a1**

Compound **a1** was prepared from 4-(*N*,*N*-diethylamino)salicylaldehyde **2** (0.07 g, 0.38 mmol), 4-amidino-1,2-phenylenediamine hydrochloride **4** (0.07 g, 0.38 mmol), and *p*-benzoquinone (0.04 g, 0.38 mmol) in absolute ethanol (5 mL) after refluxing for 4 h. The mixture was worked up as previously described to obtain 0.11 g (84%) of beige powder; m.p. >300 °C; ^1^H NMR (400 MHz, DMSO-*d_6_*) (δ/ppm): 13.29 (bs, 1H, NH_benzimidazole_), 9.30 (s, 2H, NH_amidine_), 9.05 (s, 2H, NH_amidine_), 8.06 (d, *J* = 8.7 Hz, 3H, H_arom_.), 7.63 (s, 1H, H_arom_.), 7.62 (d, *J* = 7.8 Hz, 1H, H_arom_.), 6.90 (d, *J* = 8.9 Hz, 2H, H_arom_.), 3.44 (q, *J* = 6.0 Hz, 4H, CH_2_), 1.12 (t, *J* = 6.1 Hz, 6H, CH_3_); ^13^C NMR (100 MHz, DMSO-*d_6_*) (δ/ppm): 166.2, 149.1, 128.5, 121.3 (2C), 120.2, 115.2, 111.0 (3C), 43,7 (2C), 12.4 (2C). Found: C, 63.18; H, 6.99; N, 19.63; Calc. for C_19_H_26_ClN_5_: C, 63.41; H, 7.28, N, 19.46%.

**5(6)-amidino-2-[4-(*N*,*N*-diethylamino)-2-hydroxyphenyl]benzimidazole hydrochloride a2**

Compound **a2** was prepared from 4-(*N*,*N*-diethylamino)salicylaldehyde **3** (0.10 g, 0.52 mmol), 4-amidino-1,2-phenylenediamine hydrochloride **4** (0.10 g, 0.52 mmol), and *p*-benzoquinone (0.06 g, 0.52 mmol) in absolute ethanol (2.5 mL) after refluxing for 4 h. The mixture was worked up as previouslydescribed to obtain 0.03 g (16%) of green powder; m.p. >300 °C; ^1^H NMR (400 MHz, DMSO-*d_6_*) (δ/ppm): 13.47 (bs, 2H, NH_benzimidazole_, OH), 9.36 (s, 2H, NH_amidine_), 8.96 (s, 2H, NH_amidine_), 8.08 (s, 1H, H_arom_.), 7.92 (d, *J* = 8.7 Hz, 2H, H_arom_.), 7.82 (d, *J* = 8.1 Hz, 2H, H_arom_.), 7.73 (d, *J* = 7.9 Hz, 2H, H_arom_.), 6.49 (d, *J* = 8.1 Hz, 2H, H_arom_.), 6.31 (s, 2H, H_arom_.), 3.43 (d, *J* = 6.5 Hz, 4H, CH_2_), 1.15 (t, *J* = 6.3 Hz, 6H, CH_3_); ^13^C NMR (100 MHz, DMSO-*d_6_*) (δ/ppm): 166.2, 160.3, 129.7, 123.7, 113.6, 105.2, 97.7, 44.5, 13.0. Found: C, 60.05; H, 6.19 Cl, 9.83; N, 19.48; O, 4.45. Calc. for C_19_H_17_ClN_4_S: C, 60.08; H, 6.16; Cl, 9.85; N, 19.46; O, 4.45%.

**5(6)-(2-imidazolinyl)-2-[4-(*N*,*N*-dimethylamino)phenyl]benzimidazole hydrochloride b1**

Compound **b1** was prepared from 4-(*N*,*N*-dimethylamino)benzaldehyde **1** (0.10 g, 0.67 mmol), 4-(2-imidazolinyl)-1,2-phenylenediamine hydrochloride **5** (0.14 g, 0.67 mmol), and *p*-benzoquinone (0.07 g, 0.67 mmol) in absolute ethanol (3 mL) after refluxing for 4 h. The mixture was worked up as previouslydescribed to obtain 0.19 g (85%) of light brown powder; m.p. >300 °C; ^1^H NMR (300 MHz, DMSO-*d_6_*) (δ/ppm): 13.44 (bs, 1H, NH_benzimidazole_), 13.39 (bs, 1H, NH_benzimidazole_), 10.60 (s, 4H, NH_amidine_), 8.30–8.21 (m, 2H, H_arom_.), 8.10 (s, 4H, H_arom_.), 7.81 (d, 2H, *J* = 8.6 Hz, H_arom_.), 7.79–7.61 (m, 2H, H_arom_.), 6.85 (d, 4H, *J* = 9.0 Hz, H_arom_.), 4.01 (s, 8H, CH_2_), 3.02 (s, 12H, CH_3_); ^13^C NMR (75 MHz, DMSO-*d_6_*) (δ/ppm): 166.0, 152.3, 128.7, 122.4, 116.3, 112.2, 44.7. Found: C, 63.96; H, 6.68; N, 19.75. Calc. for C_19_H_24_ClN_5_: C, 63.77; H, 6.76; N, 19.57%.

**5(6)-(2-imidazolinyl)-2-(phenanthren-9-yl)-1*H*-benzo[d]imidazole b2**

Compound **b2** was prepared from phenantrene-9-carbaldehyde **7** (0.15 g, 0.73 mmol), 4-(2-imidazolinyl)-1,2-phenylenediamine hydrochloride **5** (0.16 g, 0.73 mmol), and *p*-benzoquinone (0.08 g, 0.73 mmol) in absolute ethanol (6 mL) after refluxing for 4 h. The mixture was worked up as previouslydescribed to obtain 0.15 g (52%) of light violet powder; m.p. >300 °C; ^1^H NMR (300 MHz, DMSO-*d_6_*) (δ/ppm): 13.99 (bs, 1H, NH_benzimidazole_), 13.88 (bs, 1H, NH_benzimidazole_), 10.82 (s, 2H, NH_amidine_), 10.73 (s, 2H, NH_amidine_), 9.08 (d, 2H, *J* = 8.3 Hz, H_arom_.), 9.00 (d, 2H, *J* = 8.4 Hz, H_arom_.), 8.95 (d, 2H, *J* = 8.3 Hz, H_arom_.), 8.61 (s, 1H, H_arom_.), 8.53 (s, 1H, H_arom_.), 8.51 (s, 1H, H_arom_.), 8.44 (s, 1H, H_arom_.), 8.13 (d, 2H, *J* = 7.3 Hz, H_arom_.), 8.03–7.93 (m, 2H, H_arom_.), 7.91 (d, 1H, *J* = 7.6 Hz, H_arom_.), 7.85 (dt, 2H, *J_1_* = 7.0 Hz, *J_2_* = 1.5 Hz, H_arom_.), 7.81 (dt, 2H, *J_1_* = 7.1 Hz, *J_2_* = 1.4 Hz, H_arom_.), 7.78–7.75 (m, 4H, H_arom_.), 4.05 (s, 8H, CH_2_); ^13^C NMR (75 MHz, DMSO-*d_6_*) (δ/ppm): 165.7, 159.8, 155.8, 154.7, 143.9, 139.0, 131.0, 130.7, 129.8 (2C), 128.0, 127.7 (4C), 127.4, 127.3, 123.8, 123.7 (2C), 123.6 (3C), 123.4, 122.3, 121.0, 119.8, 115.9, 115.6, 113.3, 112.7, 44.6 (4C). Found: C, 72.45; H, 4.65; N, 13.85. Calc. for C_24_H_19_ClN_4_: C, 72.27; H, 4.80; N, 14.05%.

**5(6)-(1,4,5,6-tetrahydropyrimidin-2-yl)-2-[4-(*N*,*N*-dimethylamino)phenyl]benzimidazole hydrochloride c1**

Compound **c1** was prepared from 4-(*N*,*N*-dimethylamino)benzaldehyde **1** (0.07 g, 0.44 mmol), 4-(1,4,5,6-tetrahydropyrimidin-2-yl)-1,2-phenylenediamine hydrochloride **6** (0.103 g, 0.44 mmol), and *p*-benzoquinone (0.05 g, 0.44 mmol) in absolute ethanol (2 mL) after refluxing for 4 h. The mixture was worked up as previouslydescribed to obtain 0.06 g (37%) of light brown powder; m.p. >300 °C; ^1^H NMR (400 MHz, DMSO-*d_6_*) (δ/ppm): 13.38 (s, 1H, NH_benzimidazole_), 9.95 (s, 2H, NH_amidine_), 8.10 (d, 2H, *J* = 7.9 Hz, H_arom,_), 7.95 (s, 1H, H_arom_.), 7.68 (d, 1H, *J* = 6.6 Hz, H_arom_.), 7.52 (d, 1H, *J* = 7.9 Hz, H_arom_.), 6.85 (d, 2H, *J* = 8.2 Hz, H_arom_.), 3.52 (s, 4H, CH_2_), 3.02 (s, 6H, CH_3_), 2.01 (bs, 2H, CH_2_); ^13^C NMR (75 MHz, DMSO-*d_6_*) (δ/ppm): 160.2, 152.1, 128.7, 121.6, 121.2, 116.7, 112.2, 40.2, 39.3, 18.5. Found: C, 64.80; H, 6.96; N, 18.68. Calc. for C_20_H_26_ClN_5_: C, 64.59; H, 7.05; Cl, N, 18.83%.

**2-[4-(*N*,*N*-diethylamino)]-5(6)-(1,4,5,6-terahydropyrimidin-2-yl)benzimidazole hydrochloride c2**

Compound **c2** was prepared from 4-(*N*,*N*-diethylamino)benzaldehyde **2** (0.05 g, 0.31 mmol), 4-(1,4,5,6-tetrahydropyrimidin-2-yl)-1,2-phenylenediamine hydrochloride **6** (0.07 g, 0.31 mmol), and *p*-benzoquinone (0.03 g, 0.31 mmol) in absolute ethanol (5 mL) after refluxing for 4 h. The mixture was worked up as previouslydescribed to obtain 0.08 g (69%) of beige powder; m.p. >300 °C; ^1^H NMR (400 MHz, DMSO-*d_6_*) (δ/ppm): 13.18 (s, 1H, NH_benzimidazole_), 9.89 (s, 2H, NH_amidine_), 8.04 (d, 2H, *J* = 7.6 Hz, H_arom,_), 7.93 (s, 1H, H_arom_.), 7.67 (s, 1H, H_arom_.), 7.49 (d, 1H, *J* = 7.8 Hz, H_arom_.), 6.82 (d, 2H, *J* = 7.8 Hz, H_arom_.), 3.47 (s, 4H, CH_2_), 3.43 (q, 4H, *J* = 5.2 Hz, CH_2_), 2.02–1.99 (m, 2H, CH_2_), 1.09 (t, 6H, *J* = 5.2 Hz, CH_3_); ^13^C NMR (75 MHz, DMSO-*d_6_*) (δ/ppm): 160.3, 149.5, 129.0 (2C), 121.6, 121.2, 115.7, 111.5 (3C), 44.2 (2C), 18.5, 12.9 (2C). Found: C, 66.25; H, 7.36; N, 17.68. Calc. for C_22_H_30_ClN_5_: C, 66.07; H,7.56; N, 17.51%.

**2-[4-(*N*,*N*-diethylamino)-2-hydroxyphenyl]-5(6)-(1,4,5,6-terahydropyrimidin-2-yl) benzimidazole hydrochloride c3**

Compound **c3** was prepared from 4-(*N*,*N*-diethylamino)salicylaldehyde **3** (0.10 g, 0.52 mmol), 4-(1,4,5,6-tetrahydropyrimidin-2-yl)-1,2-phenylenediamine hydrochloride **6** (0.10 g, 0.52 mmol), and *p*-benzoquinone (0.06 g, 0.52 mmol) in absolute ethanol (2.5 mL) after refluxing for 4 h.

The mixture was worked up as previouslydescribed to obtain 0.12 g (66%) of light purple powder; m.p. > 300 °C; ^1^H NMR (300 MHz, DMSO-*d_6_*) (δ/ppm): 13.49 (s, 1H, NH_benzimidazole_), 13.38 (s, 1H, NH_benzimidazole_), 12.68 (s, 1H, OH), 12.60 (s, 1H, OH), 9.96 (s, 2H, NH_amidine_), 9.91 (s, 2H, NH_amidine_), 8.06–7.85 (m, 4H, H_arom_.), 7.83–7.62 (m, 2H, H_arom_.), 7.56 (d, 2H, *J* = 8.4 Hz, H_arom_.), 6.40 (d, 2H, *J* = 7.6 Hz, H_arom_.), 6.21 (s, 2H, H_arom_.), 3.52 (s, 8H, CH_2_), 3.40 (bs, 8H, CH_2_), 2.01 (s, 4H, CH_2_), 1.14 (s, 12H, CH_3_); ^13^C NMR (100 MHz, DMSO-*d_6_*) (δ/ppm): 160.6, 160.4, 160.2, 156.4, 155.8, 151.4, 151.3, 145.3, 141.6, 137.3, 133.6, 128.7, 128.5, 122.3, 122.1, 121.8, 117.5, 117.0, 116.1, 111.7, 111.1, 104.4, 100.2, 100.1, 98.1, 44.3, 18.4, 12.8. Found: C, 63.28; H,7.70; N, 16.70; O, 3.98. Calc. for C_22_H_30_ClN_5_O: C, 63.52; H, 7.52; N, 16.84%.

### 3.2. Biochemistry

#### 3.2.1. Heterologous Expression and Purification of Recombinant Human DPP III

C-terminally truncated human DPP III was expressed and purified as described previously [52]. In brief, C-terminal HIS-tagged recombinant human DPP III was expressed *in E. coli* BL21-CodonPlus (DE3)-RIL cells, Agilent (Santa Clara, CA, USA). Overexpression was induced by 0.25 mM isopropyl-b-D-1-thiogalactopyranoside (IPTG, Fermentas), and the cells were grown for 20 h at 18 °C and 130 rpm. Obtained bacterial cells were lysed by a combination of lysozyme lysis and sonication, then treated with DNase I and centrifuged at 4000× *g* for 15 min to precipitate the cell debris. The lysate was purified by affinity chromatography on a Ni-NTA column (5 mL prepacked His-trap FF) GE Healthcare (Chicago, IL, USA) using a buffer system with 50 mM Tris- HCl, pH = 8.0, 300 mM NaCl, and increasing imidazole concentrations: 10 mM for lysis, 20 mM for wash, and 300 mM for elution buffer. All hDPP III active fractions were pooled and incubated with TEV protease to remove the HIS-tag. Human DPP III was recovered using flow-through affinity chromatography (TEV protease is His-tagged), and additionally purified on a 16/60 Superdex-200 gel-filtration column GE Healthcare (Chicago, IL, USA). Fractions with hDPP III were desalted on PD-10 columns GE Healthcare (Chicago, IL, USA). Purified protein in 20 mM Tris-HCl buffer pH = 7.4 was stored at −80 °C until use.

#### 3.2.2. Enzyme Activity Assay and IC_50_ Determination

Recombinant hDPP III (0.75 nM) was preincubated with benzimidazole derivatives (30 µM) first for 10 min at 25 °C and then for 5 min at 37 °C in 50 mM Tris-HCl buffer, pH 7.4. The enzymatic reaction was started with Arg-Arg-2-naphthylamide (40 μM) as a substrate, and after the 15 min incubation at 37 °C in a water bath, the reaction was stopped, and the absorbance was measured using the spectrophotometric method described by Abramić et al. [3]. All benzimidazole derivatives were prepared daily in dimethyl sulfoxide, and the resulting stock solutions (4 mM) were transparent, without turbidity or solid residue. The final concentration of DMSO, Sigma-Aldrich ( St. Louis, MO, USA) in the reaction mixture did not exceed 1% and did not adversely affect enzyme activity. Before the enzyme assay, stock solutions were diluted with 50 mM Tris-HCl buffer, pH 7.4.

The percentage of enzyme inhibition (% inh.) was calculated by comparing enzymatic activity in the absence (control activity) and presence (inhibited activity) of the inhibitor using the following formula:

% inh. = [(control activity − inhibited activity)/(control activity)] × 100

Compounds that showed the strongest inhibition (% inh. ≥ 90) were subjected to further IC_50_ determination, defined as the inhibitor concentration required to reduce enzyme activity by 50% under assay conditions. The percentage of enzyme inhibition under at least seven different concentration gradients was imported into GraphPad Prism 10.4.1 software to calculate IC_50_ values of the compounds using nonlinear regression analysis from the mean inhibitory value of three replicates [57]. The relative occupancy of inhibition for compounds with the same substituent at position 6 of the benzimidazole core was defined as the sum of their % inh., expressed relative to their maximum achievable inhibition, which was set at 100.

### 3.3. Computational Study of Human DPP III Inhibitors

#### 3.3.1. QSAR

The drawing and optimization of benzimidazole structures were performed using Avogadro 1.2.0 (University of Pittsburgh, Pittsburgh, PA, USA) [58]. Initially, the structures were optimized with the MM+ molecular mechanics force field, followed by geometry optimization using the AM1 semi-empirical method [59]. This process utilized the Polak–Ribiere algorithm and continued until the root-mean-square gradient (RMS) reached 0.1 kcal/(Å mol) [60]. Molecular descriptors for the three-dimensional optimized structures of the compounds were generated using the Parameter Client (Virtual Computational Chemistry Laboratory), which is an electronic version of the Dragon 5.4 software available at https://vcclab.org/lab/pclient/ (accessed on 20 January 2025) [61]. Descriptors with more than 85% constant values, values equal to zero, or those that were highly inter-correlated (R > 90%) were eliminated from further calculations using QSARINS 2.2.4 (University of Insubria, Varese, Italy, 2019) [62]. After this reduction process, the final number of descriptors was 450. The values for hDPPIII inhibition percentage were transformed into logarithmic values and used as the response variable. The compounds were split into the training and test set by activity sampling [63]. Compounds were ranked by their inhibitory activities and then divided into five groups of the approximately same size. One or two compounds from each group were selected randomly and assigned to the test set. A genetic algorithm (GA) was employed to generate the best model using four descriptors in the multiple linear regression (MLR) equation via QSARINS 2.2.4. The model was then evaluated through methods of internal validation, including “leave-one-out” (LOO) cross-validation and the Y-scrambling method, as well as external validation on the test set [41,42,64]. The applicability domain of the QSAR model was assessed using Williams plots, which plot residuals against the leverage of compounds. This analysis helped identify potential outliers and compounds that fell outside the warning leverage (h*) of the model. The warning leverage is defined as 3*p’*/*n*, where *n* represents the number of training compounds and *p’* denotes the number of adjustable parameters in the model [65]. Compounds with standardized residual values higher than two standard deviation units were categorized as outliers.

#### 3.3.2. Docking

The starting model for molecular docking was built using the semi-closed form of ligand-free hDPP III, as described earlier by 101 ns long molecular dynamic simulations of the enzyme open form (PDB ID: 3FVY) [66]. The semi-closed form of hDPP III was used because it has been proven to be the most preferable enzyme form in an aqueous solution [67]. 3D structures of ligands were drawn and optimized as described in Section 3.3.1. MGL Tools 1.5.6 was employed to prepare the pdbqt format for molecular docking. For the receptor molecule, Kollman united atom charges and polar hydrogen atoms were added, while Gasteiger charges and hydrogen atoms were added for the ligand molecules. AutoDockVina 1.1.2 was used to search for the best ligand position at the enzyme active site [68]. The docking site for the ligand on hDPP III was defined by a grid box with the dimensions 30.0 Å^3^, centred on the enzyme active site. Specifically, the centre set was at x = −1.45, y = 2.82, and z = 2.94, i.e., ≈ 5 Å from the Cα atom of ASN381 (part of the lower β sheet) of hDPP III towards the central enzyme cleft. Docking simulation was done with a grid box spacing of 0.275 Å, exhaustiveness value was 8, and the number of genetic algorithm runs was set to 9. The complex with the best docking score and orientation was chosen, and the non-covalent interactions of the ligand–receptor complex were analysed and visualized using Discovery Studio Visualizer, version 20.1.0.19295 [69], and Visual Molecular Dynamics, version 1.9.4a53 software [70].

## 4. Conclusions

In this paper, we report the inhibition profiles of 36 amidino-substituted benzimidazole derivatives against human DPP III, combining in vitro experiments with in silico approaches. The biochemical assay revealed that the moiety associated with strong inhibitory activity features a structure that includes 2,2′-bithiophene, 4-trifluoromethylphenyl, 4-(*N*,*N*-diethylamino)phenyl, and 2,3,4-trihydroxyphenyl at position 2 of the benzimidazole core. Furthermore, compounds containing substituents at position 5(6) of the benzimidazole core—2-imidazolinyl > unsubstituted amidine > 2-tetrahydropyrimidine (ranked according to their effectiveness—were also shown to achieve better inhibitory activity. Compound **b13** (5(6)-(4,5-Dihydro-1H-imidazol-3-ium-2-yl)-2-(2,3,4-trihydroxyphenyl)benzimidazole chloride) was found to be the most potent inhibitory molecule, with an IC_50_ value of 1.03 ± 0.01 µM. The obtained QSAR model indicated that amidino-substituted benzimidazole derivatives should have electronegative substituents contributing to their polarity, spaced at topological distances of 3 and/or 4 for enhanced hDPP III inhibition. The presence of sp3-hybridized carbon atoms, however, has a negative effect on inhibition. According to these guidelines, several improved analogues of the experimentally best benzimidazole amidines were proposed. A molecular docking study of six highly potent benzimidazole derivatives showed that their inhibitory activity arises from multiple interactions between aryl and amidine groups and the amino acid residues located within the peptide binding subsite, as well as those crucial for the catalytic activity of hDPP III. Docking studies also indicate that the binding mechanism of the proposed compounds in the enzyme active subsite exhibit similar patterns to those of their parent compounds. However, in the proposed compounds, a notable increase in interactions between the benzimidazole core and the enzyme was observed.

## Figures and Tables

**Table 1:** Inhibition of human DPP III hydrolytic activity by amidino-substituted benzimidazole derivatives.

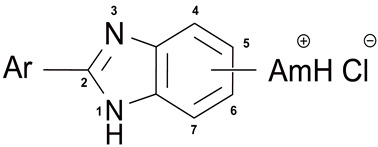					
Cpd	Am	Ar	% inh. exp. *	log % inh. exp.	log % inh. calc. **
**a1**		4-(*N*,*N*-diethylamino) phenyl	62.9 ± 0.8	1.80	2.07
**a2**	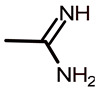	4-(*N*,*N*-diethylamino)-2-hydroxyphenyl	100.0 ± 0.0	2.00	2.17
**a3**		benzo[*b*]thiophene-2-yl	59.4 ± 1.4	1.77	1.60
**a4**		2,2′-bithiophenyl	99.6 ± 0.8	2.00	1.95
**a5**		4-cyanophenyl	67.8 ± 1.2	1.83	1.59
**a6**		4-trifluoromethylphenyl	98.7 ± 0.0	1.99	2.01
**a7**		3*H*-benzimidazolyl	21.6 ± 0.2	1.33	1.38
**a8**		naphthalen-1-yl	38.7 ± 0.8	1.59	1.51
**a9**		quinolin-2-yl	82.9 ± 0.2	1.92	1.73
**a10**		2-hydroxyphenyl	24.3 ± 0.2	1.39	1.32
**a11**		2,4-dihydroxyphenyl	14.6 ± 3.1	1.16	1.45
**a12**		2-hydroxy-4-methoxyphenyl	51.5 ± 1.3	1.71	1.65
**b1**	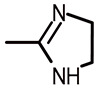	4-(*N*,*N*-dimethylamino)phenyl	52.9 ± 1.7	1.72	1.95
**b2**		phenanthren-9-yl	100.0 ± 0.0	2.00	1.97
**b3**		benzo[*b*]thiophene-2-yl	60.6 ± 2.1	1.77	1.81
**b4**		2,2′-bithiophenyl	100.0 ± 0.0	2.00	2.06
**b5**		4-cyanophenyl	34.4 ± 0.2	1.54	1.57
**b6**		4-trifluoromethylphenyl	99.4 ± 1.0	2.00	1.95
**b7**		3*H*-benzimidazolyl	51.1 ± 1.8	1.71	1.59
**b8**		naphthalen-1-yl	29.5 ± 1.2	1.47	1.71
**b9**		quinolin-2-yl	73.4 ± 2.6	1.87	1.95
**b10**		2-hydroxyphenyl	42.8 ± 3.0	1.63	1.28
**b11**		2,4-dihydroxyphenyl	41.1 ± 0.8	1.61	1.40
**b12**		2-hydroxy-4-methoxyphenyl	47.1 ± 0.9	1.67	1.59
**b13**		2,3,4-trihydroxyphenyl	100.0 ± 0.0	2.00	1.86
**c1**		4-(*N*,*N*-dimethylamino)phenyl	9.9 ± 1.3	1.00	0.98
**c2**		4-(*N*,*N*-diethylamino) phenyl	100.0 ± 0.0	2.00	1.71
**c3**	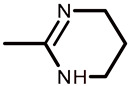	4-(*N*,*N*-diethylamino)-2-hydroxyphenyl	92.6 ± 0.5	1.97	1.83
**c4**		2,2′-bithiophenyl	99.1 ± 0.8	2.00	1.70
**c5**		4-cyanophenyl	0.1 ± 0.2	−1.00 (0.00 ***)	0.60
**c6**		4-trifluoromethylphenyl	15.5 ± 0.9	1.19	1.02
**c7**		3*H*-benzimidazolyl	29.8 ± 3.8	1.47	1.24
**c8**		phenanthren-9-yl	51.7 ± 0.2	1.71	1.73
**c9**		phenyl	2.1 ± 0.7	0.32	0.20
**c10**		naphthalene-2-yl	8.0 ± 0.5	0.90	1.22
**c11**		benzo[*b*]thiophene-2-yl	28.9 ± 3.1	1.46	1.47

* At the 30 μmol L^–1^ concentration of the compound (data represent the average values of three determinations ± standard deviation); ** Calculated by quantitative structure–activity relationship (QSAR) equation: log % hDPP III inh. = −3.90 + 0.06 *Rww* + 1.25 *Mats3e* + 3.81 *BELe4 −* 0.94 *nCs*; *** Value used for the purposes of generating QSAR models.

**Table 2:** Statistical results of the best QSAR model for human DPP III inhibition.

Statistical Parameters	QSAR Model Values
*N_training set_*	29
*N_test set_*	7 (a3, a9, b3, b5, b12, c1, c4)
*R* ^2^	0.82
*R* ^2^ *_adj_*	0.79
*s*	0.23
*F*	26.93
*K_xx_*	0.26
*ΔK*	0.10
*RMSE*	0.21
*MAE*	0.16
*CCC*	0.90
*Q* ^2^ *_LOO_*	0.72
*Q* ^2^ *_LMO_*	0.69
*RMSE_cv_*	0.26
*MAE_cv_*	0.19
*CCC_cv_*	0.85
*R* ^2^ *_Yscr_*	0.15
*Q* ^2^ *_Yscr_*	−0.26
*R* ^2^ *_ext_*	0.87
*CCC_ext_*	0.86
*RMSE_ext_*	0.15
*MAE_ext_*	0.12
*Q* ^2^ *_F1_*	0.77
*Q* ^2^ *_F2_*	0.75
*Q* ^2^ *_F3_*	0.90
*r* ^2^ *_m_* *aver*	0.75
*r* ^2^ *_m_* *diff*	0.13

*R*^2^ (coefficient of determination); *R*^2^*_adj_* (adjusted coefficient of determination); *s* (standard deviation of regression); *F* (Fisher ratio); *K_xx_* (multivariate correlation index); Δ*K* (global correlation among descriptors); *RMSE* (root-mean-square error of the training set); *MAE* (mean absolute error of the training set); *CCC* (concordance correlation coefficient of the training set); *Q*^2^*_LOO_* (the leave-one-out cross-validated explained variance); *Q*^2^*_LMO_* (the leave-many-out cross-validated explained variance); *RMSE_cv_* (root-mean-square error of the training set determined through the cross validated method; *MAE_cv_* (mean absolute error of the internal validation set); *CCC_cv_* (concordance correlation coefficient test set using cross validation); *R*^2^*_Yscr_* (Y-scramble correlation coefficients); *Q*^2^*_Yscr_* (Y-scramble cross-validation coefficients); *RMSE_ext_* (root-mean-square error of the external validation set); *MAE_ext_* (mean absolute error of the external validation set); *R*^2^*_ext_* (coefficient of determination of validation set); *Q*^2^*_F__n_* (predictive squared correlation coefficients); *CCC_ext_* (concordance correlation coefficient of the test set); *r*^2^*_m_* average (average value of squared correlation coefficients between the observed and (leave-one-out) predicted values of the compounds with and without intercept); *r*^2^*_m_* difference (absolute difference between the observed and leave-one-out predicted values of the compounds with and without intercept.

## Data Availability

Data are contained within the article or Supplementary Materials.

## Supplementary Materials

The following supporting information can be downloaded at: https://www.mdpi.com/article/10.3390/ijms26083899/s1.
