# Supplementary material for: New Amidino-Substituted Benzimidazole Derivatives as Human Dipeptidyl Peptidase III Inhibitors: Synthesis, In Vitro Evaluation, QSAR, and Molecular Docking Studies
Source: Int J Mol Sci. 2025 Apr 20;26(8):3899. doi: 10.3390/ijms26083899 (PMC12027992; doi:10.3390/ijms26083899)
Supplement: Supplementary file 1 [file ijms-26-03899-s001.zip › ijms-3580055-supplementary.pdf]

Supplementary materials

New amidino-substituted benzimidazole derivatives as human dipeptidyl peptidase III inhibitors: Synthesis, *in vitro* evaluation, QSAR and molecular docking studies

Dejan Agić[1*], Maja Karnaš Babić[1], Marijana Hranjec[2], Domagoj Šubarić[1], Zrinka Karačić[3] Marija Abramić[4]

1. Faculty of Agrobiotechnical Sciences Osijek, Josip Juraj Strossmayer University of Osijek, 31000 Osijek, Croatia; dejan.agic@fazos.hr (D.A.); maja.karnas@fazos.hr (M.K.B.); domagoj.subaric@fazos.hr (D.Š.)
2. Department of Organic Chemistry, Faculty of Chemical Engineering and Technology, University of Zagreb, 10000 Zagreb, Croatia; mhranjec@fkit.hr (M.H.)
3. Division of Molecular Biology, Ruđer Bošković Institute, 10000 Zagreb, Croatia; zrinka.karacic@irb.hr (Z.K.)
4. Divison of Organic Chemistry and Biochemistry, Ruđer Bošković Institute, 10000 Zagreb, Croatia; marija.abramic@irb.hr (M.A.)

*Correspondence: dejan.agic@fazos.hr (D.A.)

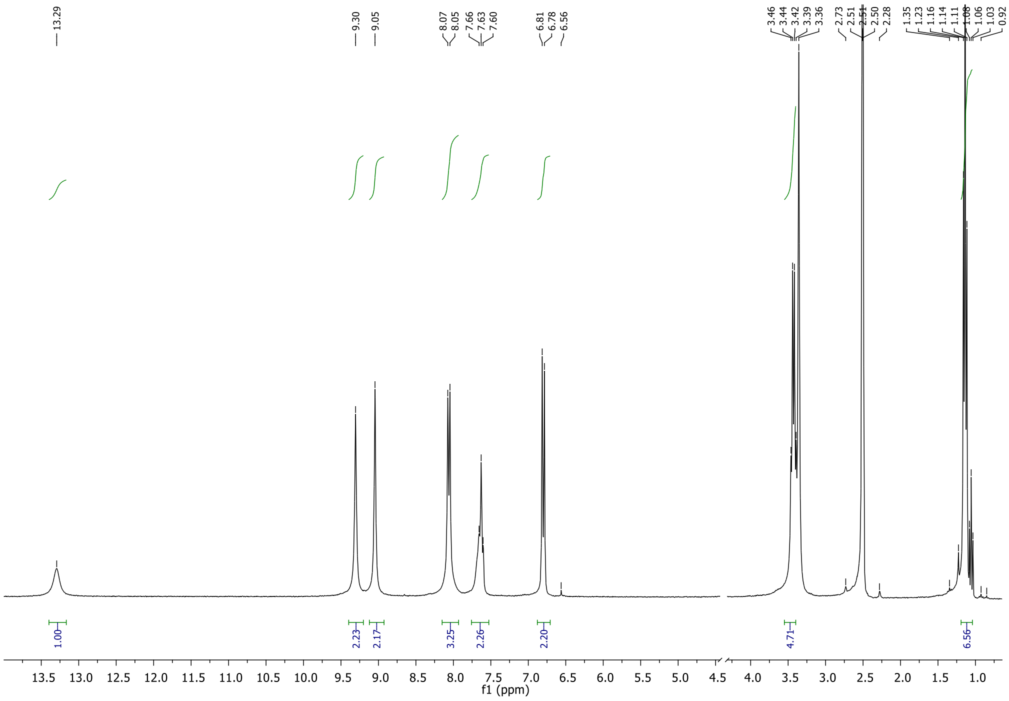

Figure S1: ^{1}H NMR spectra of 5(6)-amidino-2-4-(*N*,*N*-diethylamino)benzimidazole hydrochloride **a1**

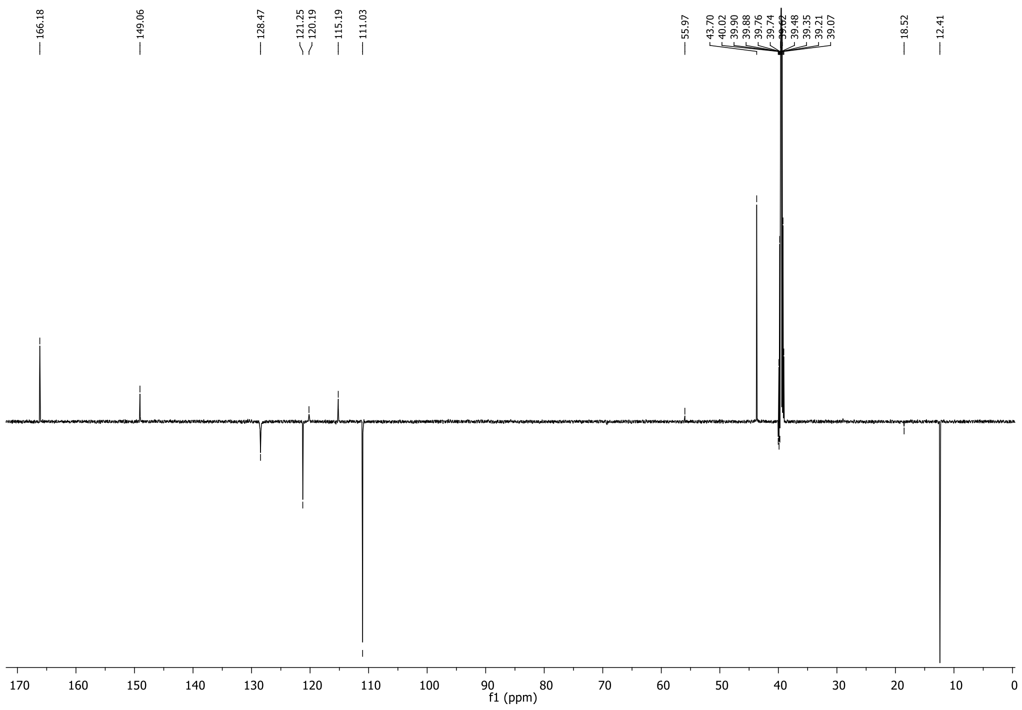

Figure S2: ^{13}C NMR spectra of 5(6)-amidino-2-4-(*N,N*-diethylamino)benzimidazole hydrochloride **a1**

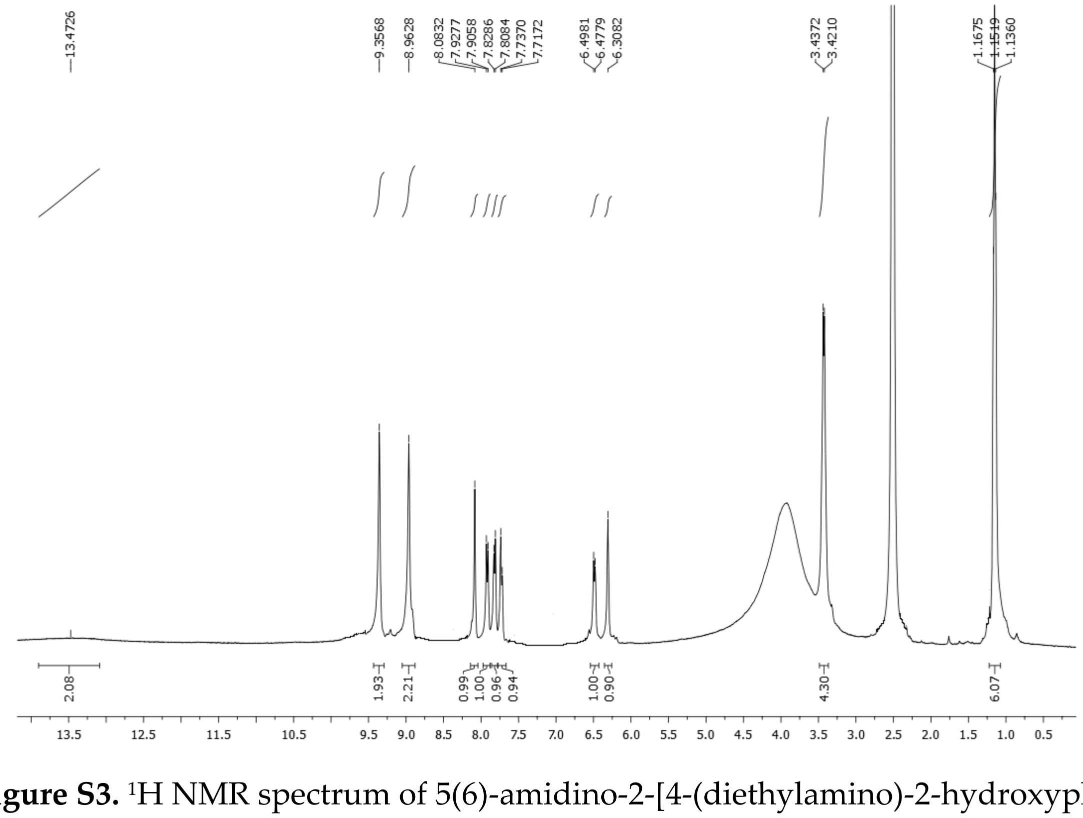

Figure S3. ^{1}H NMR spectrum of 5(6)-amidino-2-[4-(diethylamino)-2-hydroxyphenyl] benzimidazole hydrochloride **a2**

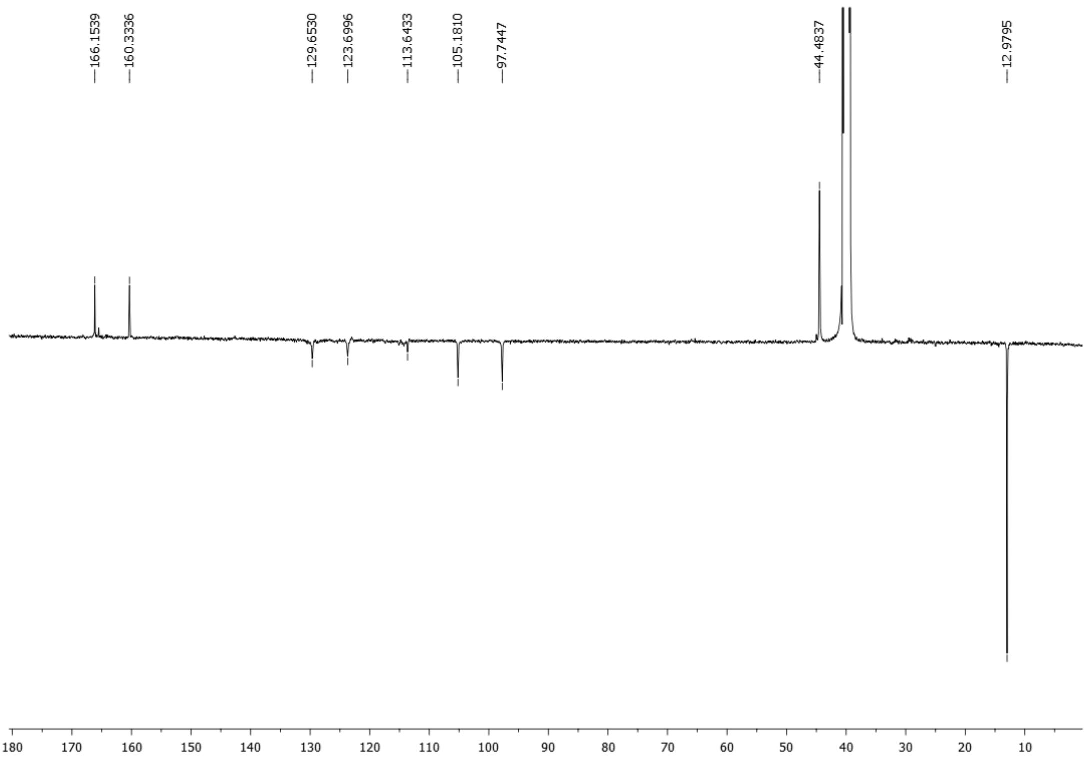

Figure S4. ^{13}C NMR spectrum of 5(6)-amidino-2-[4-(diethylamino)-2-hydroxyphenyl] benzimidazole hydrochloride **a2**

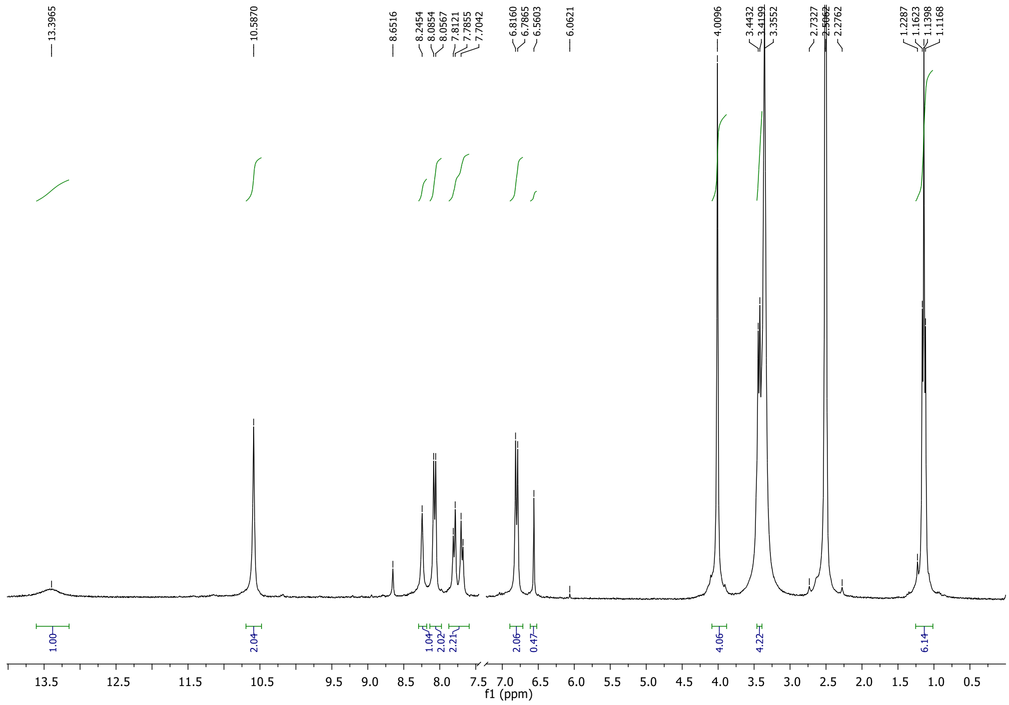

Figure S5. ^{1}H NMR spectrum of 5(6)-(2-imidazolinyl)-2-[4-(*N,N*-dimethylamino) phenyl]benzimidazole hydrochloride **b1**

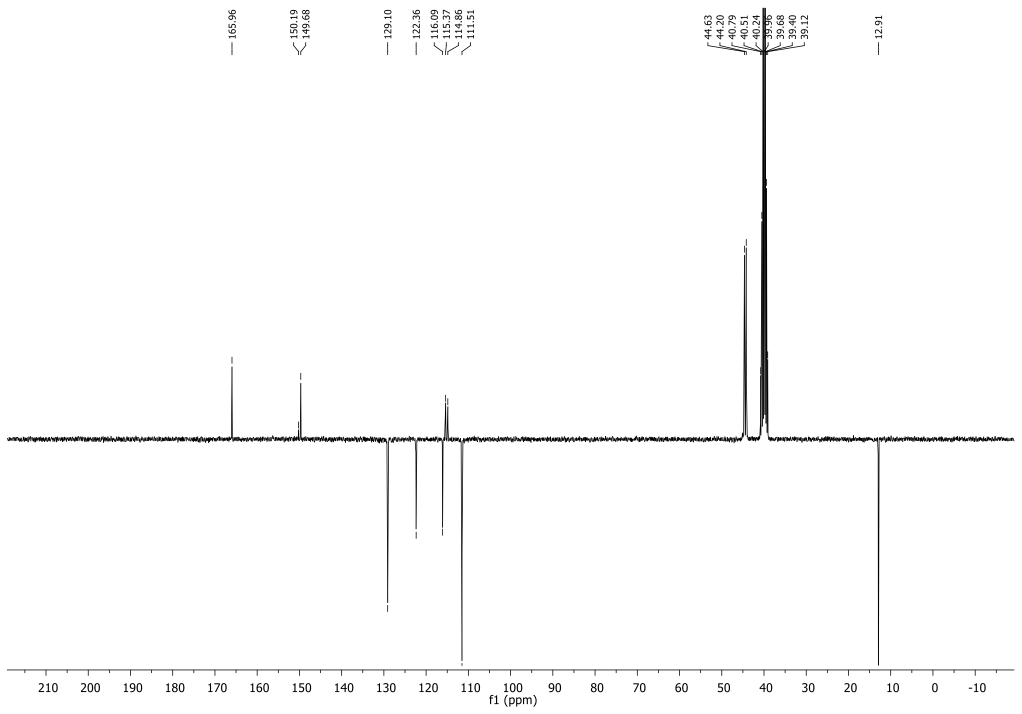

Figure S6. ^{13}C NMR spectrum of 5(6)-(2-imidazolinyl)-2-[4-(*N,N*-dimethylamino) phenyl]benzimidazole hydrochloride **b1**

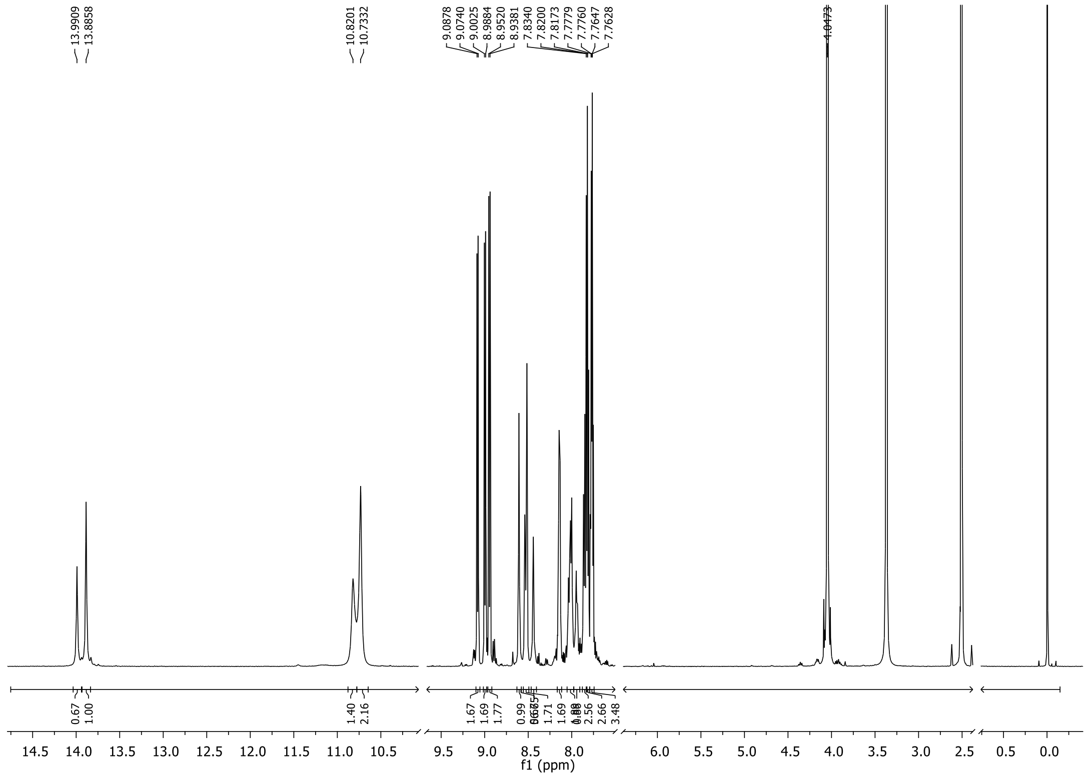

Figure S7. ^{1}H NMR spectrum of 5(6)-(2-imidazolinyl)-2-(phenanthren-9-yl)-1*H*-benzo[d]imidazole **b2**

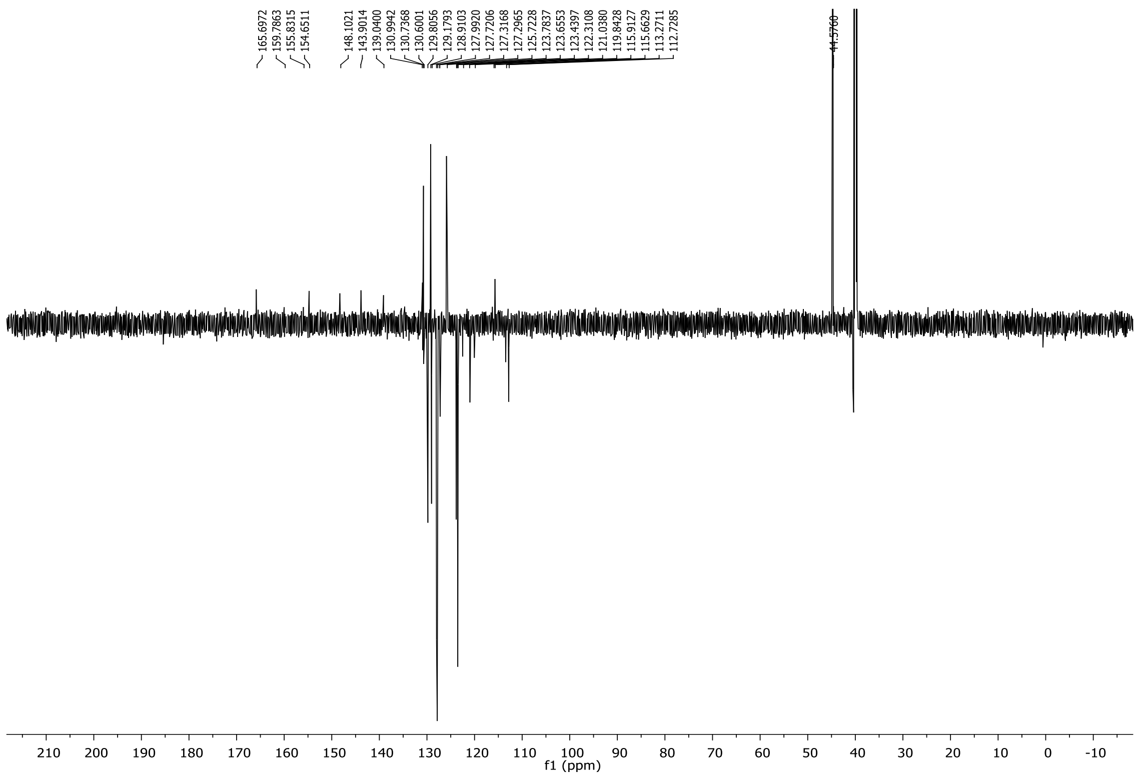

Figure S8. ^{13}C NMR spectrum of 5(6)-(2-imidazolinyl)-2-(phenanthren-9-yl)-1*H*-benzo[d]imidazole **b2**

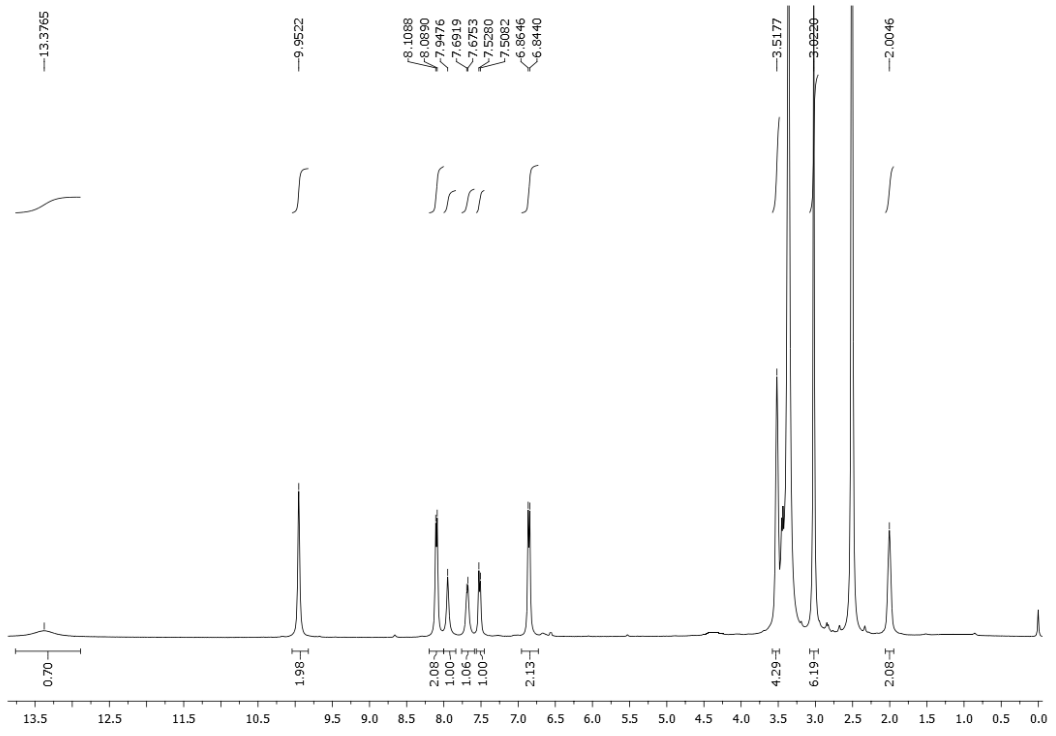

Figure S9. ^{1}H NMR spectrum of 2-[4-(dimethylamino)phenyle]-5(6)-(1,4,5,6-tetrahydro-pyrimidin-2-yl)benzimidazole hydrochloride **c1**

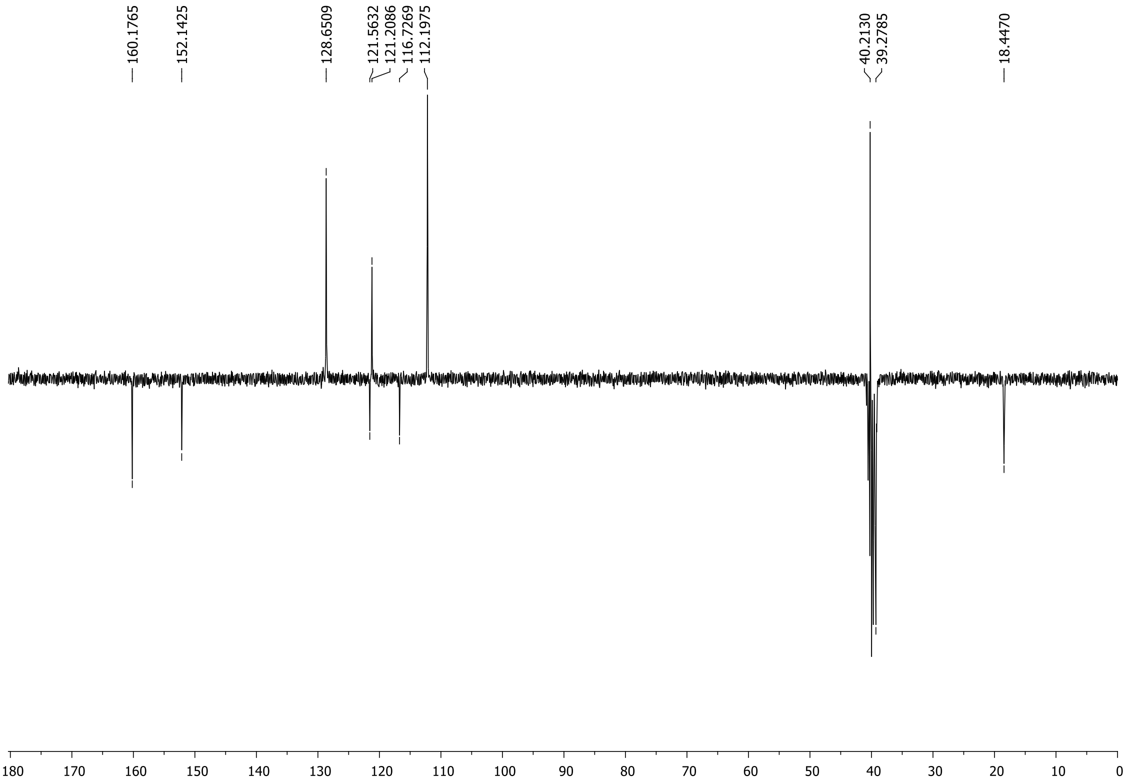

Figure S10. ^{13}C NMR spectrum of 2-[4-(dimethylamino)phenyle]-5(6)-(1,4,5,6-tetrahydro-pyrimidin-2-yl)benzimidazole hydrochloride **c1**

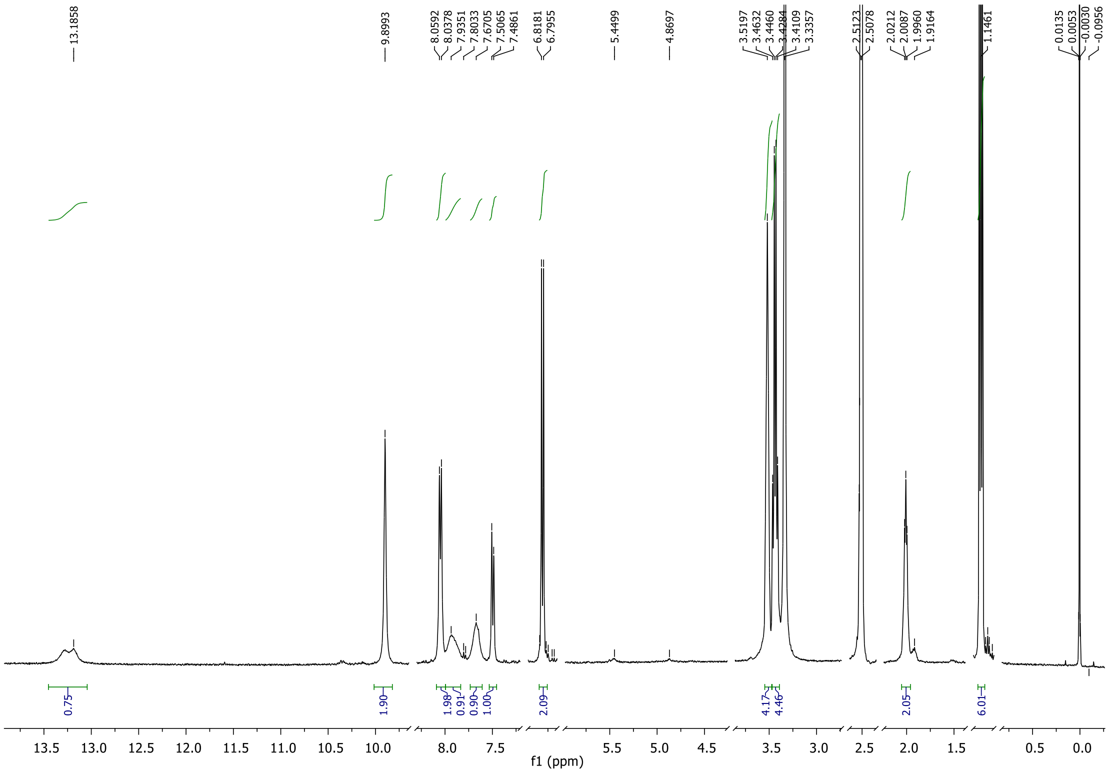

Figure S11. ^{1}H NMR spectrum of 2-[4-(*N,N*-diethylamino)]-5(6)-(1,4,5,6-terahydro-pyrimidin-2-yl)benzimidazole hydrochloride **c2**

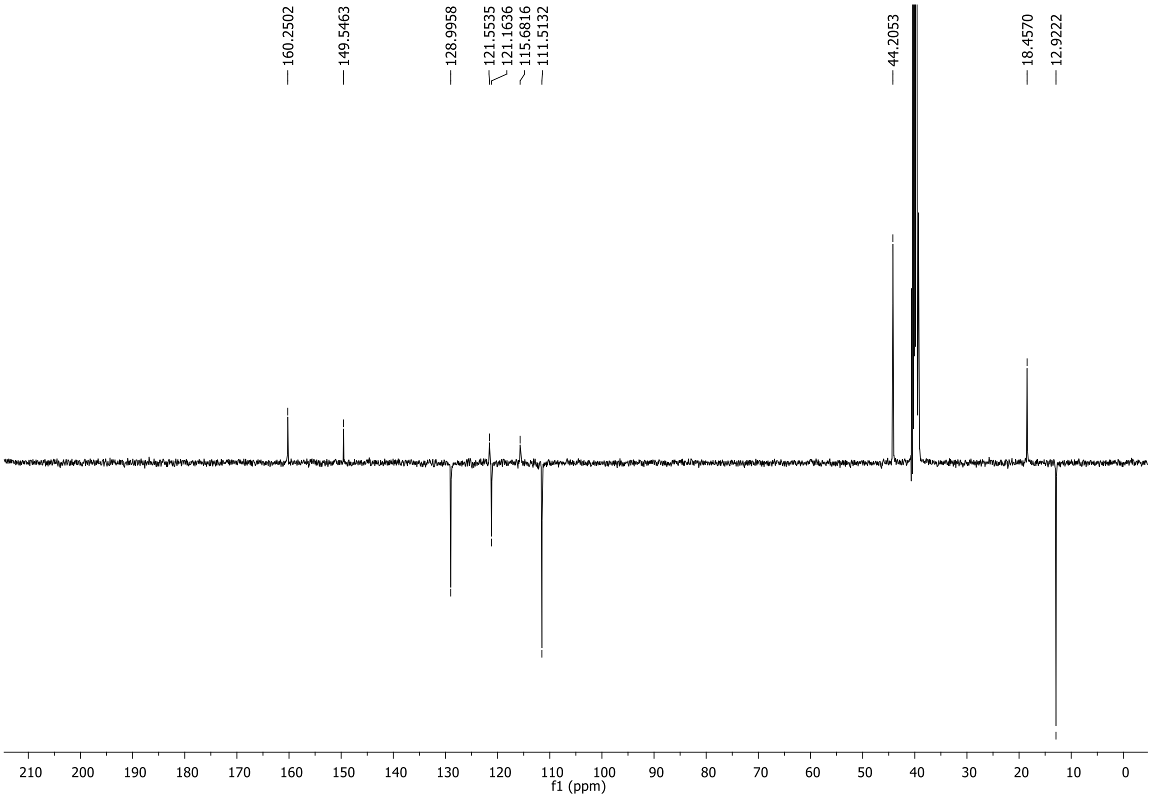

Figure S12. ^{13}C NMR spectrum of 2-[4-(*N,N*-diethylamino)]-5(6)-(1,4,5,6-terahydro-pyrimidin-2-yl)benzimidazole hydrochloride **c2**

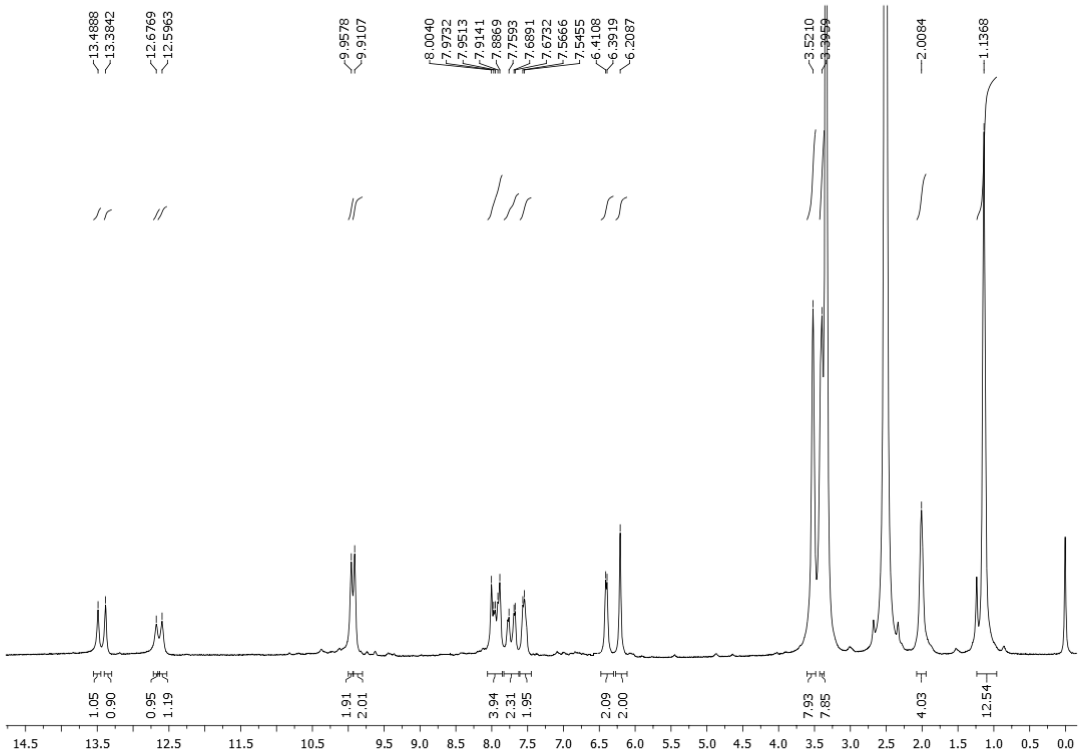

Figure S13. ^{1}H NMR spectrum of 2-[4-(diethylamino)-2-hydroxyphenyl]-5(6)-(1,4,5,6-terahydropyrimidin-2-yl)benzimidazole hydrochloride **c3**

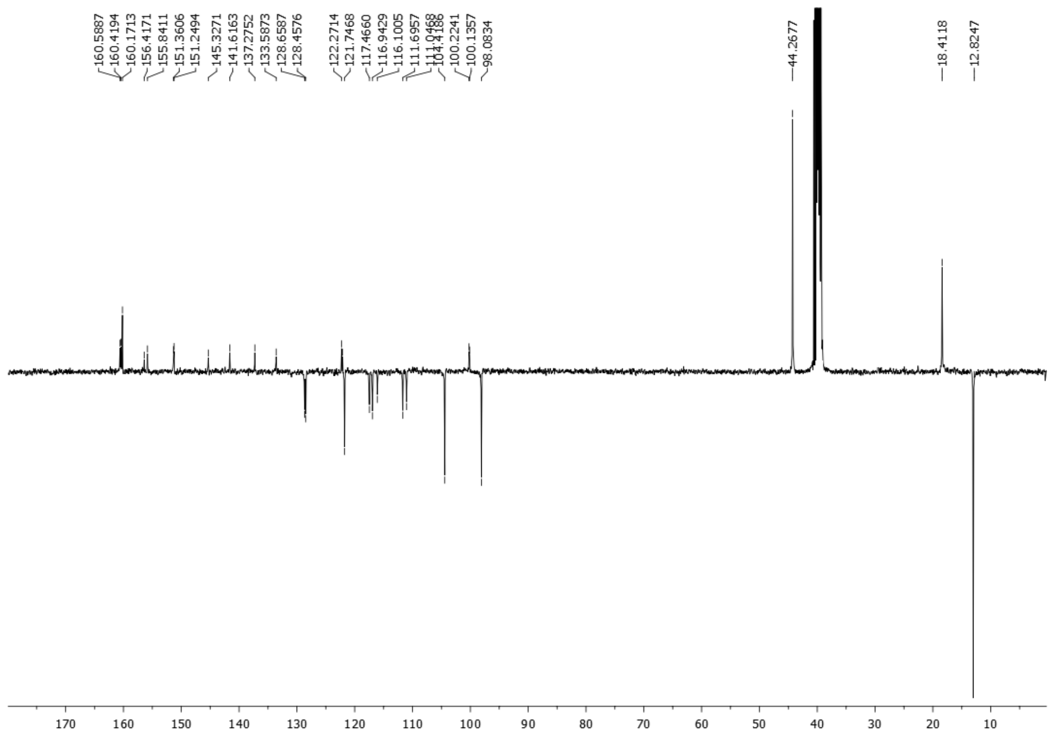

Figure S14. ^{13}C NMR spectrum of 2-[4-(diethylamino)-2-hydroxyphenyl]-5(6)-(1,4,5,6-terahydropyrimidin-2-yl)benzimidazole hydrochloride **c**3

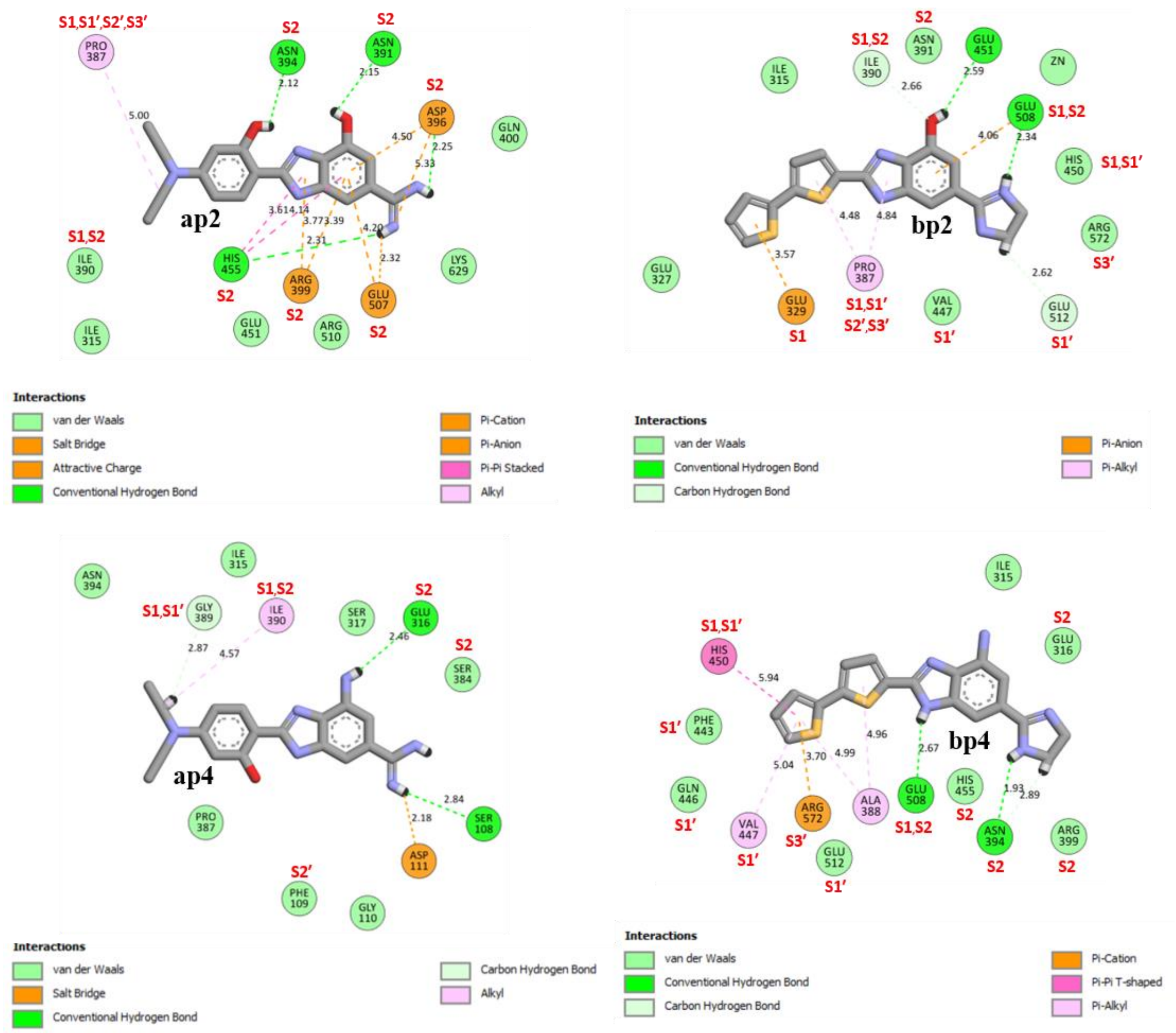

Figure S15. Binding of the four predicted benzimidazole derivatives at the human DPP III active site. 2D plot of the non-covalent interactions depicted using Discovery Studio Visualizer. Substrate binding subsites are highlighted in red.

Table S1. Values of descriptors from the best QSAR model on human DPP III inhibition for all *in vitro* tested benzimidazole derivatives.

Compound	*Rww*	*Mats3e*	*BELe4*	*nCs*
**a1**	19.204	-0.219	1.32	0
**a2**	20.846	-0.221	1.32	0
**a3**	9.656	-0.111	1.32	0
**a4**	13.674	-0.126	1.35	0
**a5**	13.078	-0.289	1.32	0
**a6**	17.544	-0.169	1.317	0
**a7**	9.64	-0.284	1.32	0
**a8**	9.688	-0.185	1.32	0
**a9**	9.717	-0.009	1.32	0
**a10**	11.279	-0.297	1.28	0
**a11**	12.86	-0.278	1.282	0
**a12**	14.628	-0.252	1.298	0
**b1**	13.986	-0.248	1.386	0
**b2**	8.488	-0.125	1.442	0
**b3**	8.485	-0.094	1.39	0
**b4**	12.52	-0.102	1.39	0
**b5**	11.911	-0.272	1.327	0
**b6**	16.394	-0.165	1.317	0
**b7**	8.468	-0.27	1.39	0
**b8**	8.521	-0.179	1.39	0
**b9**	8.551	0.011	1.39	0
**b10**	10.101	-0.279	1.284	0
**b11**	11.692	-0.263	1.285	0
**b12**	13.47	-0.241	1.298	0
**b13**	13.267	0.018	1.285	0
**c1**	14.247	-0.283	1.386	1
**c2**	18.331	-0.245	1.497	1
**c3**	19.985	-0.232	1.497	1
**c4**	12.782	-0.149	1.553	1
**c5**	12.167	-0.306	1.327	1
**c6**	16.659	-0.168	1.317	1

**c7**	8.721	-0.302	1.552	1
**c8**	8.747	-0.158	1.631	1
**c9**	8.874	-0.323	1.281	1
**c10**	8.806	-0.226	1.52	1
**c11**	8.738	-0.139	1.557	1

Table S2. Correlation matrix (with correlation coefficient values *R*) for descriptors used in model equation.

**Descriptor**	***Rww***	***Mats3e***	***BELe4***	***nCs***
Rww	1.000			
Mats3e	-0.132	1.000		
BELe4	-0.183	0.112	1.000	
nCs	0.031	-0.228	0.645	1.000

Table S3. Values of descriptors and calculated log % inhibition for the proposed benzimidazole derivatives, according to the QSAR model on human DPP III inhibition.

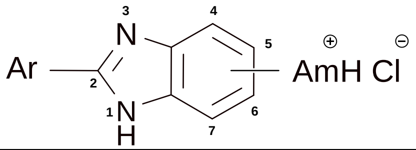

**Compound**	**Based on compound**	**R**	***Rww***	***Mats3e***	***BELe4***	***nCs***	**log % inh. calc.***
**ap1**	**a2**	5-OH	22.283	-0.237	1.32	0	2.17
**ap2**		4-OH	22.269	-0.137	1.321	0	2.30
**ap3**		5-NH_2	22.283	-0.243	1.321	0	2.17
**ap4**		4-NH_2	22.269	-0.189	1.328	0	2.26
**ap5**	**a4**	5-OH	15.085	-0.214	1.35	0	1.88
**ap6**		4-OH	15.074	-0.033	1.35	0	2.11
**ap7**		5-NH_2	15.085	-0.201	1.35	0	1.90
**ap8**		4-NH_2	15.074	-0.073	1.35	0	2.06
**bp1**	**b4**	5-OH	13.942	-0.195	1.39	0	1.99
**bp2**		4-OH	13.932	-0.011	1.39	0	2.22
**bp3**		5-NH_2	13.942	-0.177	1.39	0	2.01

**bp4**		4-NH_2	13.932	-0.046	1.393	0	2.19
**ap9**	**a6**	5-OH	18.964	-0.200	1.317	0	2.01
**ap10**		4-OH	18.951	-0.162	1.317	0	2.05
**ap11**		5-NH_2	18.964	-0.177	1.32	0	2.05
**ap12**		4-NH_2	18.951	-0.165	1.318	0	2.05
**bp5**	**b6**	5-OH	17.825	-0.195	1.318	0	1.95
**bp6**		4-OH	17.813	-0.155	1.318	0	2.00
**bp7**		5-NH_2	17.825	-0.173	1.32	0	1.98
**bp8**		4-NH_2	17.813	-0.160	1.325	0	2.02
**bp9**	**b13**	5-OH	14.685	-0.081	1.285	0	1.78
**bp10**		4-OH	14.675	-0.017	1.286	0	1.86
**bp11**		5-NH_2	14.685	-0.020	1.287	0	1.86
**bp12**		4-NH_2	14.675	0.006	1.299	0	1.94

* Calculated by quantitative structure-activity relationship (QSAR) equation: log % hDPP III inh.= -3.90 + 0.06*Rww* + 1.25*Mats3e* + 3.81*BELe4* - 0.94*nCs*

Table S4. Binding affinity of benzimidazole derivatives with human DPP III calculated by AutoDock Vina.

Compounds	Binding affinity (kcal mol^{-1})	Compounds	Binding affinity (kcal mol^{-1})	Compounds	Binding affinity (kcal mol^{-1})
**a1**	-7.1	**b1**	-7.9	**c1**	-7.8
**a2**	-7.8	**b2**	-9.4	**c2**	-8.0
**a3**	-7.7	**b3**	-8.6	**c3**	-8.1
**a4**	-7.5	**b4**	-8.0	**c4**	-8.3
**a5**	-7.3	**b5**	-8.0	**c5**	-8.0
**a6**	-7.7	**b6**	-8.2	**c6**	-8.3
**a7**	-7.9	**b7**	-8.3	**c7**	-8.5
**a8**	-8.2	**b8**	-9.2	**c8**	-10.1
**a9**	-8.0	**b9**	-8.8	**c9**	-7.7
**a10**	-7.3	**b10**	-8.1	**c10**	-9.0
**a11**	-7.0	**b11**	-8.2	**c11**	-8.5
**a12**	-7.2	**b12**	-8.4		
		**b13**	-9.0		

Table S5. The binding affinity, type, and number of interactions formed by different structural components of the six most potent benzimidazole derivatives with human DPP III.

	Compounds																	
	**a2**			**a4**			**a6**			**b4**			**b6**			**b13**		
Binding affinity (kcal mol^{-1})	-7.8			-7.5			-7.7			-8.0			-8.2			-9.0		
Interactions	Ar	Bc	Am	Ar	Bc	Am	Ar	Bc	Am	Ar	Bc	Am	Ar	Bc	Am	Ar	Bc	Am
Hydrogen bond	2	-	1	-	-	2	-	2	1	-	-	1	4	-	2	4	-	-
Attractive charge	-	-	2	-	-	2	-	-	2	-	-	-	-	-	-	-	-	-
Van der Waals	5	1	3	2	-	3	1	1	1	4	3	1	-	1	1	2	1	4
Hydrophobic bonds*	2	3	-	1	2	-	3	-	-	3	-	-	-	3	-	1	3	2
Sum	9	4	6	3	2	7	4	3	4	7	3	2	4	4	3	7	4	6
TOTAL	19			12			11			12			11			17		

Ar-aryl group; Bc-benzimidazole core; Am-amidino group; *amide-π stacked, π-alkyl, π-sulfur, π -π T-shaped, alkyl

Table S6. The number of interactions between the peptide binding subsites of human DPP III and different structural components of the six most potent benzimidazole

	Compounds																	
Subsites*	**a2**			**a4**			**a6**			**b4**			**b6**			**b13**		
	Ar	Bc	Am	Ar	Bc	Am	Ar	Bc	Am	Ar	Bc	Am	Ar	Bc	Am	Ar	Bc	Am
S2	1	1	2	-	1	1	2	3	2	-	3	2	-	1	1	-	1	3
S1	2	1	2	-	2	3	2	-	-	1	1	-	1	2	3	1	2	2
S1'	-	-	1	-	1	2	1	-	-	1	-	-	-	1	2	-	1	1
S2'	-	-	-	-	1	-	1	-	-	2	-	-	1	1	0	-	1	-
S3'	-	-	-	-	1	-	1	-	-	1	-	-		1	0	-	1	-
SUM	3	2	5	0	6	6	7	3	2	5	4	2	2	6	6	1	6	6
TOTAL	10			12			12			11			14			13		

derivatives.

*Subsites were defined according to Bezzera et al, 2012[8]; Ar-aryl group, Bc-benzimidazole core; Am- amidino group

Table S7. The binding affinity, type, and number of interactions formed by different structural components of the four predicted benzimidazole derivatives with human DPP III.

	Compounds											
	**ap2**			**ap4**			**bp2**			**bp4**		
Binding affinity (kcal mol^{-1})	-8.0			-7.8			-8.4			-8.6		
Interactions	Ar	Bc	Am	Ar	Bc	Am	Ar	Bc	Am	Ar	Bc	Am
Hydrogen bond	1	2	2	1	1	1	-	2	2	-	1	2
Attractive charge	-	4	2	-	-	1	-	-	-	-	-	-
Van der Waals	2	2	2	3	3	2	2	2	4	3	2	2
Hydrophobic bonds*	1	2	-	1	-	-	2	2	-	5	-	-
Sum	4	10	6	5	4	4	4	6	6	8	3	4
TOTAL	20			13			16			15		

Ar-aryl group; Bc-benzimidazole core; Am-amidino group; *amide-π stacked, π-alkyl, π-sulfur, π -π T-shaped, alkyl

Table S8. The number of interactions between the peptide binding subsites of human DPP III and different structural components of the four predicted benzimidazole derivatives.

Subsites*	Compounds											
	**ap2**			**ap4**			**bp2**			**bp4**		
	Ar	Bc	Am	Ar	Bc	Am	Ar	Bc	Am	Ar	Bc	Am
S2	2	5	3	2	1	-	-	3	1	-	2	3
S1	2	-	-	2	-	-	2	3	2	1	1	-
S1'	1	-	-	1	-	-	1	1	3	6	-	-
S2'	1	-	-	-	1	-	1	1	-	1	-	-
S3'	1	-	-	-	-	-	1	1	1	2	-	-
SUM	5	5	3	5	2	0	5	9	7	10	3	3
TOTAL		13			7			21			16	

*Subsites were defined according to Bezzera et al, 2012[8]; Ar-aryl group, Bc-benzimidazole core; Am-amidino group
